# CAT/SOD-Enriched *Achyranthes bidentata* nanovesicles mitigate TMJOA via ROS scavenging and JNK/FOXO1 pathway Inhibition

**DOI:** 10.1186/s12951-025-03834-9

**Published:** 2025-12-23

**Authors:** Rui Li, Zhiqing Huang, Lingyunbo Kong, Wenyi Cai, Xin Li, Chuni Hsieh, Kaihan Zheng, Chu Deng, Wei Cao, Antong Wu, Janak L. Pathak, Rong Zhang, Qingbin Zhang

**Affiliations:** 1 Department of Temporomandibular Joint, School and Hospital of Stomatology, Guangdong Engineering Research Center of Oral Restoration and Reconstruction & Guangzhou Key Laboratory of Basic and Applied Research of Oral Regenerative Medicine, Guangzhou Medical University, Guangzhou, 510180 Guangdong China; 2 Department of Stomatology, the Second Affiliated Hospital of Guangzhou Medical University, Guangzhou, China; 3 Laboratory for Myology, Department of Human Movement Sciences, Faculty of Behavioural and Movement Sciences, Vrije Universiteit Amsterdam, Amsterdam Movement Science, Amsterdam, The Netherlands

**Keywords:** Temporomandibular joint osteoarthritis, Oxidative stress, *Achyranthes bidentata*-derived nanovesicles, Macrophages, Reactive oxygen species

## Abstract

Temporomandibular joint osteoarthritis (TMJOA) is an inflammatory disorder with limited treatment options. Given the key role of oxidative stress in TMJOA progression, targeting this pathway may offer therapeutic benefits. In this study, we isolated and characterized exosome-like nanovesicles from *Achyranthes bidentata* (ABNVs), a traditional Chinese herb known for its antioxidant properties. ABNVs exhibited an average size of 134.7 nm and a negatively charged surface (-30.32 mV), and were enriched with antioxidant enzymes such as catalase (CAT) and superoxide dismutase (SOD). In a rat TMJOA model, ABNVs (1 µg/mL) effectively reduced cartilage degradation, bone damage, and synovial inflammation while maintaining high biocompatibility. Immunohistochemical analysis revealed that ABNVs suppressed M1 macrophage polarization. In vitro, ABNVs did not impair chondrogenic differentiation but mitigated chondrocyte inflammation. Mechanistically, ABNVs scavenged reactive oxygen species (ROS) and reduced macrophage inflammation through CAT/SOD-mediated ROS clearance and modulation of the JNK/FOXO1 pathway. Additionally, ABNVs indirectly enhanced chondrogenic differentiation by inhibiting M1 macrophage activation. Our findings demonstrate that ABNVs hold promise as a therapeutic strategy for TMJOA by targeting oxidative stress and inflammation via ROS scavenging and JNK/FOXO1 regulation.

**Graphical Abstract:**

**Figure:**
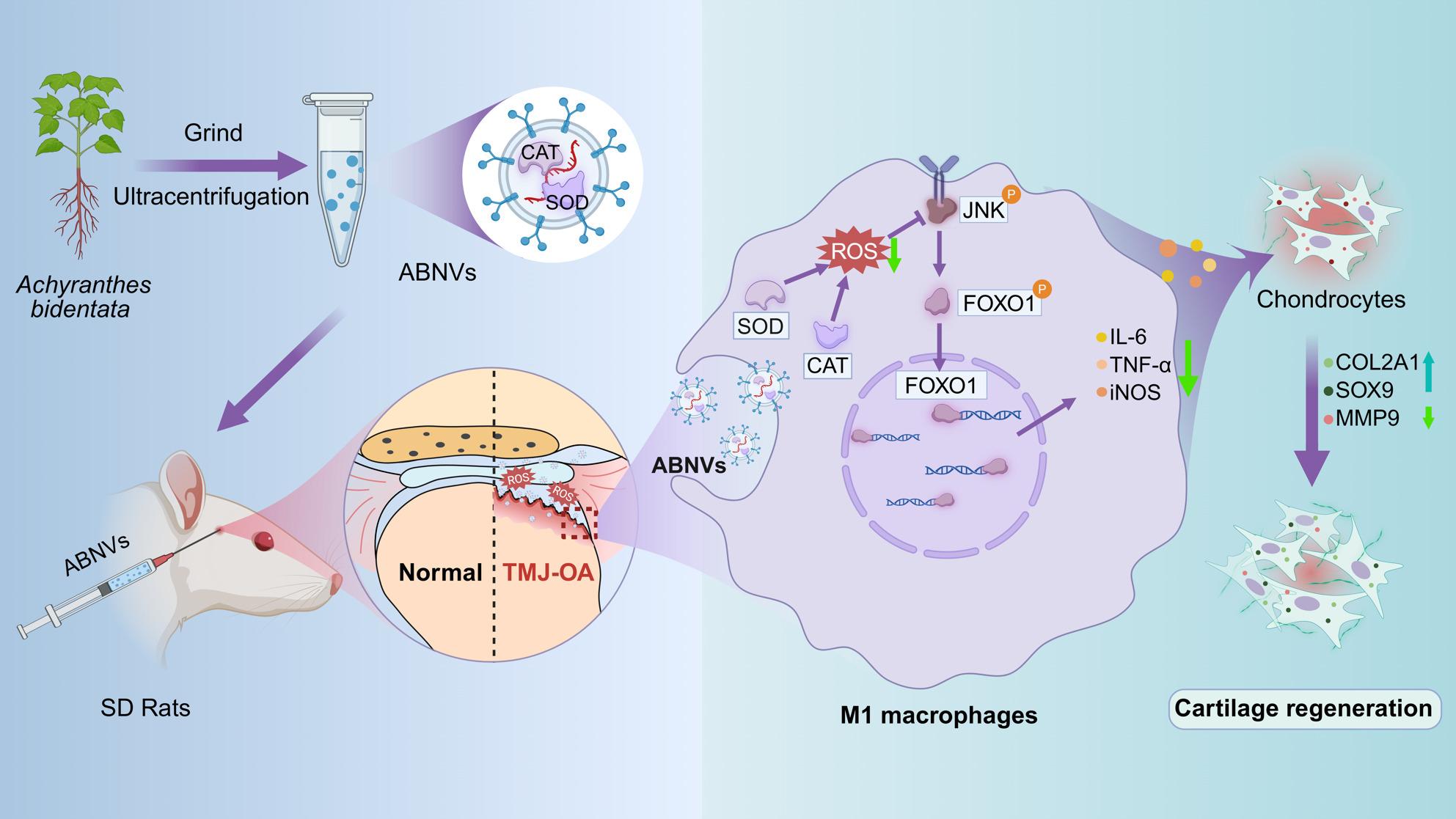
Scheme 1: The mechanism of Achyranthes bidentata-derived nanovesicles (ABNVs) ameliorates TMJOA by ROS scavenging and modulation of inflammatory levels in M1-type macrophages. This results in reduced cartilage matrix degradation and enhanced matrix synthesis in the TMJOA animal model

**Supplementary Information:**

The online version contains supplementary material available at 10.1186/s12951-025-03834-9.

## Introduction

Temporomandibular joint osteoarthritis (TMJOA) is a prevalent degenerative joint disorder that causes chronic pain, orofacial deformities, and psychological distress, including depression and anxiety [1]. The temporomandibular joint (TMJ), one of the most mechanically stressed joints in the body, is highly susceptible to damage from trauma, excessive loading, and repetitive stress, all of which contribute to TMJOA pathogenesis [2]. Epidemiological data suggest that 60–70% of adults exhibit TMJ disorder symptoms [3], with 8–16% progressing to TMJOA [4]. Current clinical management of TMJOA employs a stepwise treatment approach, initially utilizing conservative therapies such as bite splints, intra-articular injections (e.g., hyaluronic acid), and physical therapy. These treatments typically provide only temporary relief, alleviating symptoms without halting disease progression. For advanced cases, invasive surgical intervention is required, yet such procedures carry significant risks and offer unpredictable long-term outcomes [5–7]. There is currently no disease-modifying therapy capable of effectively reversing or halting the pathological progression of TMJOA, underscoring the urgent need to develop safer and more effective treatment strategies.

TMJOA is marked by articular chondrocyte apoptosis, extracellular matrix (ECM) degradation, subchondral bone remodeling, and synovial inflammation. Growing evidence highlights oxidative stress as a key driver of osteoarthritis (OA) progression [8, 9]. Under pathological conditions, excessive reactive oxygen species (ROS) accumulate in TMJ-resident cells, including chondrocytes and macrophages, triggering pro-inflammatory M1 macrophage polarization. This process amplifies the release of inflammatory mediators such as IL-1β, iNOS, TNF-α, and IL-6 [10, 11], which impair chondrocyte viability and differentiation, exacerbating cartilage destruction. Given this cascade, antioxidant-based therapies targeting ROS in macrophages and chondrocytes represent a promising approach for TMJOA treatment.

*Achyranthes bidentata* (AB), known as “Niu Xi” in Traditional Chinese Medicine, is a tropical/subtropical herb with demonstrated efficacy in osteoarthritis (OA) management. Studies indicate AB extracts promote chondrocyte proliferation, protect cartilage integrity, and reduce inflammation while exhibiting potent antioxidant activity [12–14]. However, clinical translation of traditional herbal extracts is limited by poor bioavailability, stability, and tolerability [15]. Medicinal plant-derived exosome-like nanovesicles (MPENs) offer a solution. These natural nanocarriers deliver bioactive miRNAs, lipids, and proteins with high cellular uptake efficiency [16]. Emerging research shows MPENs from turmeric, tomato, and spinach can alleviate OA through immunomodulation, anti-inflammatory effects, and cartilage regeneration [17–20]. Their superior biocompatibility, stability, low immunogenicity, and scalable production make MPENs ideal therapeutic candidates [21]. However, the potential of MPENs derived from Achyranthes bidentata, a herb with recognized efficacy in OA, remains largely unexplored for TMJOA treatment. Given the central role of oxidative stress and inflammation in TMJOA pathogenesis, we hypothesized that *Achyranthes bidentata*-derived nanovesicles (ABNVs) could serve as a novel and potent nanotherapy by leveraging their innate antioxidant enzyme cargo to target ROS accumulation and inflammatory responses in the joint simultaneously. Building on these advantages, we propose ABNVs as a next-generation TMJOA treatment.

In this study, we successfully isolated ABNVs through ultracentrifugation and systematically evaluated their therapeutic potential in OA. Our results demonstrate that ABNVs: (1) significantly attenuated cartilage degradation and subchondral bone destruction in a TMJOA rat model; (2) effectively suppressed synovial inflammation while maintaining excellent biocompatibility; and (3) outperformed hyaluronic acid, the current clinical standard for TMJOA treatment. Mechanistic studies revealed that ABNVs are enriched with antioxidant enzymes (SOD and CAT), which confer dual therapeutic effects by simultaneously scavenging ROS and modulating the JNK/FOXO1 signaling pathway, thereby mitigating M1 macrophage polarization and subsequent inflammatory cascades.

## Materials and methods

Detailed information for antibodies (Table S1) and primers (Table S2) is provided in the supplementary materials.

### Animal

All animal procedures were approved by the Ethics Committee of Guangdong HUA WEI Testing Co., Ltd. (Approval No. 2024060013). All rats were obtained from the Guangdong Medical Laboratory Animal Center and housed in specific pathogen-free (SPF) barrier facilities.

### Isolation of ABNVs

Fresh AB root was purchased from Sichuan, China. Briefly, the root of AB was gently washed twice with deionized water and then processed using a juice extractor. The extracted AB juice was sequentially centrifuged at 2,000 × g for 30 min and 10,000 × g for 60 min at 4℃ to remove large particles, fibers, and dead cells from the supernatant. Then, using a fixed-angle gravity rotor ultracentrifuge (Beckman Optima L-90 K, UAS), the supernatant was centrifuged twice at 100,000 × g for 70 min at 4℃. The resulting pellet was resuspended in 500 µL of phosphate-buffered saline (PBS) and filtered through a 0.22 μm filter. The final ABNVs were aliquoted and stored at − 80℃ for further experiments. The protein concentration of ABNVs was determined using a BCA assay kit (Beyotime, #P0010, China).

### Cell culture

Primary condylar chondrocytes were isolated from the temporomandibular joint (TMJ) of 4-week-old female Sprague-Dawley (SD) rats. The condylar cartilage was minced into small pieces, washed three times with PBS, and digested with 0.2% collagenase II (Sigma-Aldrich, USA) at 37℃ for 6 h. The digested tissue was filtered through a 40 μm nylon mesh strainer (Corning, NY, USA) to obtain single cells. RAW264.7 macrophages (National Collection of Authenticated Cell Cultures, Shanghai, China) were used as a macrophage model. Both chondrocytes and RAW264.7 macrophages were cultured as previously described [22]. Cells were passaged at 80% confluence, and second-passage (P2) chondrocytes were used for in vitro experiments.

### LPS, IL-1β, and ABNVs treatment

RAW264.7 macrophages were seeded in six-well plates and divided into three experimental groups: control, LPS, and LPS + ABNVs. The control group was maintained under standard culture conditions without treatment. In the LPS group, RAW264.7 macrophages were treated with 100 ng/mL *E. coli* LPS serotype O111:B4 (Sigma-Aldrich, USA) for 24 h. For the LPS + ABNVs group, cells were first stimulated with LPS for 24 h, after which the LPS-containing medium was replaced, and the cells were treated with ABNVs for an additional 24 h.

Similarly, condylar chondrocytes were divided into three groups: control, IL-1β, and IL-1β + ABNVs. In the IL-1β group, chondrocytes were treated with IL-1β (PeproTech, #400-01B, USA) for 24 h. For the IL-1β + ABNVs group, cells were first exposed to IL-1β for 24 h, after which the IL-1β-containing medium was replaced, and the cells were treated with ABNVs for an additional 24 h. The control group remained untreated throughout the experiment.

### Conditioned medium (CM) collection

After treating macrophages with either LPS or LPS + ABNVs, the original medium was replaced with fresh serum-free medium. Following 12 h of incubation, the culture supernatants were collected and centrifuged at 1,000 × g for 5 min. The supernatants were then mixed with DMEM/F12 medium at a 1:1 ratio and supplemented with 10% fetal bovine serum (FBS) and 1% penicillin-streptomycin (PS) to prepare the CM. The CM was designated as Ctrl-CM (from untreated macrophages), LPS-CM (from M1-type macrophages), and LPS + ABNVs-CM (from ABNVs co-cultured with M1-type macrophages) [22]. Condylar chondrocytes were subsequently cultured in these CM for 7 days for further experiments.

### Animal experiment

Eight-week-old female specific-pathogen-free (SPF) Sprague-Dawley (SD) rats were randomly divided into four groups (6 rats per group): sham, osteoarthritis (OA) + normal saline (NS), OA + hyaluronic acid (HA), and OA + ABNVs. HA is derived from Sculptra^®^ medical hyaluronic acid gel, with a weight-average molecular weight ≥ 850 kDa. The TMJOA model was established as described in our previous study [23]. Each experimental subject received a bilateral intratibial injection of 0.5 mg monoiodoacetate (MIA) (Sigma-Aldrich, USA) in 50 µL PBS, targeting the anterosuperior compartment of the TMJ. The sham group received 50 µL PBS. After 2 weeks, the sham group underwent further needling. The OA + NS group received 50 µL of NS, the OA + HA group received 50 µL of HA, and the OA + ABNVs group received intra-articular injections of 50 µL ABNVs (1 µg/µL) weekly for 4 weeks. Six weeks after the start of the experiment, the rats were euthanized via a rapid intraperitoneal overdose of sodium pentobarbital. Peripheral blood was collected from the exposed abdominal aorta into anticoagulation tubes for routine blood tests. Following euthanasia, blood, heart, liver, spleen, lungs, kidneys, and bilateral TMJs were harvested for subsequent experiments.

### Cell counting Kit-8 (CCK-8) assay

Cells were cultured in 96-well plates and exposed to various treatment regimens for 24, 48, or 72 h. After optimal growth, 10 µL of CCK-8 (Dojindo, #CK04, Japan) was added to each well. After 2 h of incubation, the OD450 was measured using a microplate reader (Thermo Fisher Scientific, Waltham, MA, USA).

### Uptake of ABNVs by RAW264.7

ABNVs were labeled with PKH26 red fluorescent dye (MedChemExpress) and co-cultured with RAW264.7 for 24 h. Cells were fixed in 4% paraformaldehyde for 15 min, rinsed three times with PBS, and stained with 4’,6-diamidino-2-phenylindole (DAPI) (Beyotime, China) for 1 h. The cell samples were observed under an inverted fluorescence microscope (Leica, Germany).

### RT-qPCR analysis

Total RNA was isolated using an RNA isolation kit (EZB, #B0004D, China). The concentration of RNA was determined using the NanoDrop 2000 (Thermo Scientific, MA, USA). A reverse transcription kit (Accurate Biotechnology, #AG11621, China) was utilized to transcribe the isolated RNA into cDNA. The primer sequences utilized are provided in Table S2.

### Western blot

Extracted protein (25 µg) from cells was loaded onto polyacrylamide gels for electrophoresis, transferred to PVDF membranes (Millipore, #IPVH00010, USA), and blocked with 5% skim milk for 2 h. Next, membranes were incubated with primary antibodies at 4℃ overnight, followed by incubation with corresponding HRP-conjugated secondary antibodies at room temperature for 1 h. Protein bands were detected using an ECL kit (Epizyme Biomedical Technology, China) and quantified, with normalization against GAPDH, using ImageJ software (National Institutes of Health, USA). The experiments were independently repeated three times. Details of the primary antibodies used for western blotting are provided in Table S1.

### Toluidine blue staining

Chondrocytes (2 × 10⁴ cells/well) seeded in 48-well plates were cultured with ABNVs or macrophage CM for 7 d. Cells were fixed with 4% paraformaldehyde for 15 min and stained with toluidine blue (Solarbio, #G3660, China). Images were taken using a light microscope (Leica Microsystems, Germany).

### Micro-CT analysis

TMJ specimens were collected and fixed with 4% paraformaldehyde for 48 h. TMJ condyles were scanned using a micro-CT (Al 0.5 mm filter; Bruker MicroCT, SkyScan1276, Germany) to observe structural changes, which were then reconstructed using NRecon software (dynamic image range 0.016000–0.058000; Bruker MicroCT, Germany). The histomorphometric parameters, including BV/TV, BS/BV, Tb.N, and Tb.Sp, were analyzed using CTAn software (threshold 80–255, Bruker MicroCT, Germany). Additionally, 3D images were reconstructed using CTVox software (Bruker MicroCT, Germany) for morphological assessment.

### Histology

TMJ samples were demineralized in 10% EDTA (pH 7.2–7.4) for 6 weeks, dehydrated, embedded in paraffin, and sectioned at 4 μm thickness. The samples were then stained with Safranin O and Fast Green (Servicebio, #G1053, China). Cartilage thickness was quantified by H&E staining, whereas proteoglycan changes in the cartilage matrix were analyzed via Safranin O staining. Osteoarthritis (OA) severity was graded using the modified Mankin scoring system.

### Immunohistochemistry

After deparaffinization and rehydration, paraffin sections underwent citrate-based antigen retrieval. Endogenous peroxidase activity was blocked by incubation with 3% hydrogen peroxide for 25 min. Sections were uniformly coated with 3% bovine serum albumin and incubated for 30 min at room temperature. After removal of the blocking solution, sections were incubated with primary antibodies (COL2A1, IL-6, Aggrecan, CD163, iNOS and MMP13) overnight at 4℃ under humidified conditions. Subsequently, horseradish peroxidase-conjugated secondary antibodies (Servicebio, 1:300, GB21303, China) were applied for 50 min at room temperature. Following three PBS washes (5 min each), color development was performed using DAB substrate (Servicebio, 1:100, GB11117, China). After rinsing with tap water, sections were restained with hematoxylin (Servicebio, #G1004, China) for 3 min, dehydrated through an ethanol series, cleared in xylene, and sealed with neutral mounting medium. Images were captured using a conventional bright-field microscope. Details of primary antibodies used for immunohistochemistry are provided in Table S1.

### Immunofluorescence staining

Chondrocytes were seeded on chamber slides (Thermo Scientific, #177402PK, USA). Cells were fixed with 4% paraformaldehyde for 15 min at RT, washed with PBS three times, permeabilized with 0.25% Triton X-100 (Servicebio, #G1204, China), and then blocked with 1% BSA at RT for 30 min. Afterward, cells were incubated with primary antibodies (MMP3, MMP9, and COL2A1) at 4℃ overnight. Following PBS washes, cells were incubated with horseradish peroxidase-conjugated goat anti-rabbit secondary antibody (Servicebio, 1:300, #GB21303, China) for 1 h at 37℃. Finally, cells were stained with antifade mounting medium containing DAPI (Beyotime, #P0131, China) and observed under a fluorescence microscope (Leica, Germany). Details of primary antibodies used for immunofluorescence are provided in Table S1.

### Intracellular ROS measurements

The intracellular ROS levels in cells were quantified using DCFH-DA (Beyotime, China) fluorescence probes. Cell samples were placed in a chamber slide and incubated with 10 µM DCFH-DA for 20 min at 37℃ in the dark. Fluorescence images were captured using a fluorescence microscope (Leica, Germany).

### Proteomic analysis

The protein expression in ABNVs was analyzed by APTBIO (Shanghai, China). The analysis process involved two stages: mass spectrometry experiments and data analysis. The mass spectrometry experiments included protein extraction, peptide digestion, and liquid chromatography-tandem mass spectrometry (LC-MS/MS) data-independent acquisition (DIA). Subsequently, bioinformatics analysis of the DIA data was performed, which included protein identification, differential expression analysis, and functional analysis.

### SOD and CAT detection

The levels of SOD and CAT in ABNVs were measured using the SOD assay kit (Beyotime, #S0051, China) and the CAT assay kit (Beyotime, #S0101S, China), respectively. All assessments were conducted following the manufacturer’s instructions.

### Trypsin digestion experiment

Incubate ABNVs with 0.25% trypsin at 37℃ for 5 min and 30 min, then terminate digestion with 4 volumes of PBS relative to the trypsin volume. Centrifuge at 100,000 g for 70 min at 4 °C to remove residual trypsin. Resuspend the pellet in PBS and determine the activity of both enzymes using the CAT and SOD activity assay kits.

### Transcriptome sequencing

Total RNA was extracted from RAW264.7 macrophages using the Trizol Plus RNA Purification Kit (Thermo Fisher Scientific, USA). RNA degradation and contamination were assessed by 1% agarose gel electrophoresis. RNA concentration and purity were measured using a Nanodrop 2000 instrument (Thermo Fisher Scientific, USA), and RNA integrity was evaluated with the RNA Nano 6000 Assay Kit on the Agilent Bioanalyzer 2100 System (Agilent Technologies, USA). Library construction and sequencing were conducted by Health Time Gene (Shenzhen, China). RNA-seq data analysis was performed as described in our previous work [24].

### IVIS imaging

ABNVs was labeled with DiR (MedChemExpress, #HY-D1048, USA) and then precipitated by ultracentrifugation at 100,000×g for 70 min. After washing with PBS, DiR-labeled ABNVs was resuspended in PBS. Free DiR and DiR-labeled ABNVs were administered intra-articularly into rat joints following MIA-induced arthritis. Fluorescence intensity within rats was detected using an In Vivo Imaging System (IVIS, PerkinElmer, USA) on Day 1 and subsequent time points.

### Statistical analysis

All data are presented as mean ± standard deviation and analyzed using GraphPad Prism 8.0 software (GraphPad, USA). Comparisons between two groups were performed using the Student’s t-test, while comparisons among more than two groups were evaluated by one-way analysis of variance (ANOVA).

## Results

### Isolation and biological properties of ABNVs

**Fig. 1:**
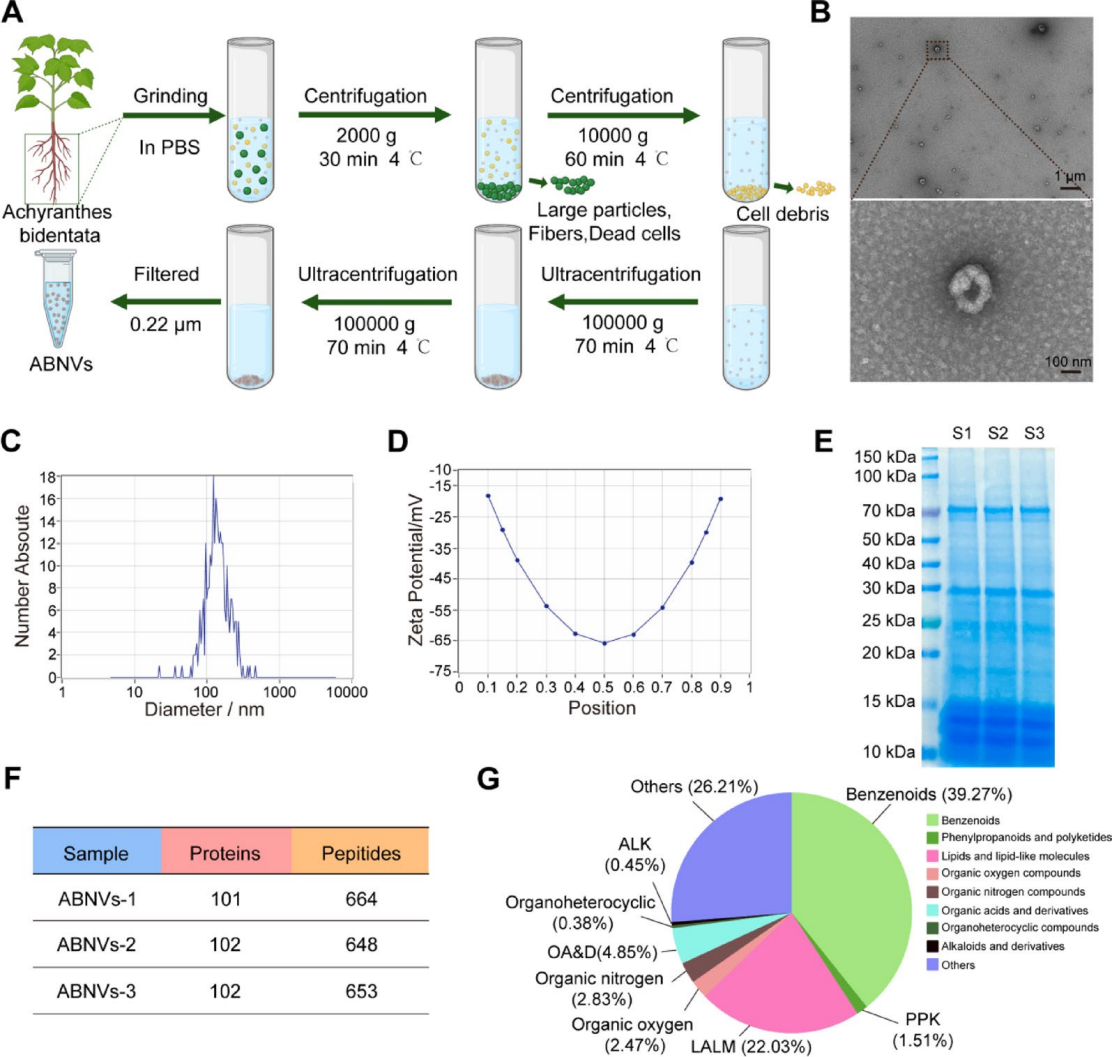
Isolation and biological properties of *Achyranthes bidentata-*derived nanovesicles (ABNVs). **(A)** The isolation procedure of ABNVs from fresh *Achyranthes bidentata* (AB) juice. **(B)** TEM images of ABNVs. **(C)** The particle size and **(D)** surface charge of ABNVs were analyzed using NTA. **(E)** ABNVs’ proteins were extracted and separated using SDS-PAGE, and then stained with Coomassie brilliant blue dye. **(F)** The total number of proteins and peptides contained in ABNVs was identified by proteomic analysis. **(G)** Non-targeted metabolomics analysis of ABNVs

ABNVs were isolated via ultracentrifugation (Fig. 1A). TEM images revealed that ABNVs exhibit a double bilayer vesicle-like structure (Fig. 1B). NTA analysis showed that ABNVs have an average particle size of 134.7 nm (Fig. 1C) and a zeta potential of −30.32 mV (Fig. 1D). SDS-PAGE identified proteins in ABNVs with molecular weights ranging from 10 to 100 kDa (Fig. 1E). Lastly, we performed proteomic, non-targeted metabolomic analyses to analyze the composition of ABNVs. A total of 102 proteins and 653 peptides were identified within ABNVs cargo (Fig. 1F). Non-targeted metabolomics to detect the presence of Benzenoids, Lipids, and lipid-like molecules, and Organic oxygen compounds in ABNVs (Fig. 1G). The endotoxin level of ABNVs was tested by the Endotoxin assay kit (Beyotime, #C0271S, China), showing no endotoxicity of ABNVs (0.03 EU/mL). These results collectively demonstrate that the isolated ABNVs possess the characteristic features of plant exosome-like nanovesicles (PENs).

### ABNVs alleviated TMJOA in rats

To establish optimal ABNVs’ concentrations for in vivo application, chondrocytes were isolated and treated with a concentration gradient (0.25–4 µg/mL) over 24–72 h (Fig. S1A-D). Notably, 4 µg/mL ABNVs markedly reduced chondrocyte viability (Fig. S1C). Following IL-1β stimulation (a standard method for modeling TMJOA in vitro [25]), chondrocytes showed upregulated ECM degradation markers (*Mmp13*, *Adamts5*) and downregulated ECM synthesis marker (*Acan*). ABNVs treatment dose-dependently suppressed catabolic gene expression while failing to reverse *Acan* downregulation (Fig. S1C-D). Based on these findings, 1 µg/mL was selected for subsequent in vivo studies.

**Fig. 2:**
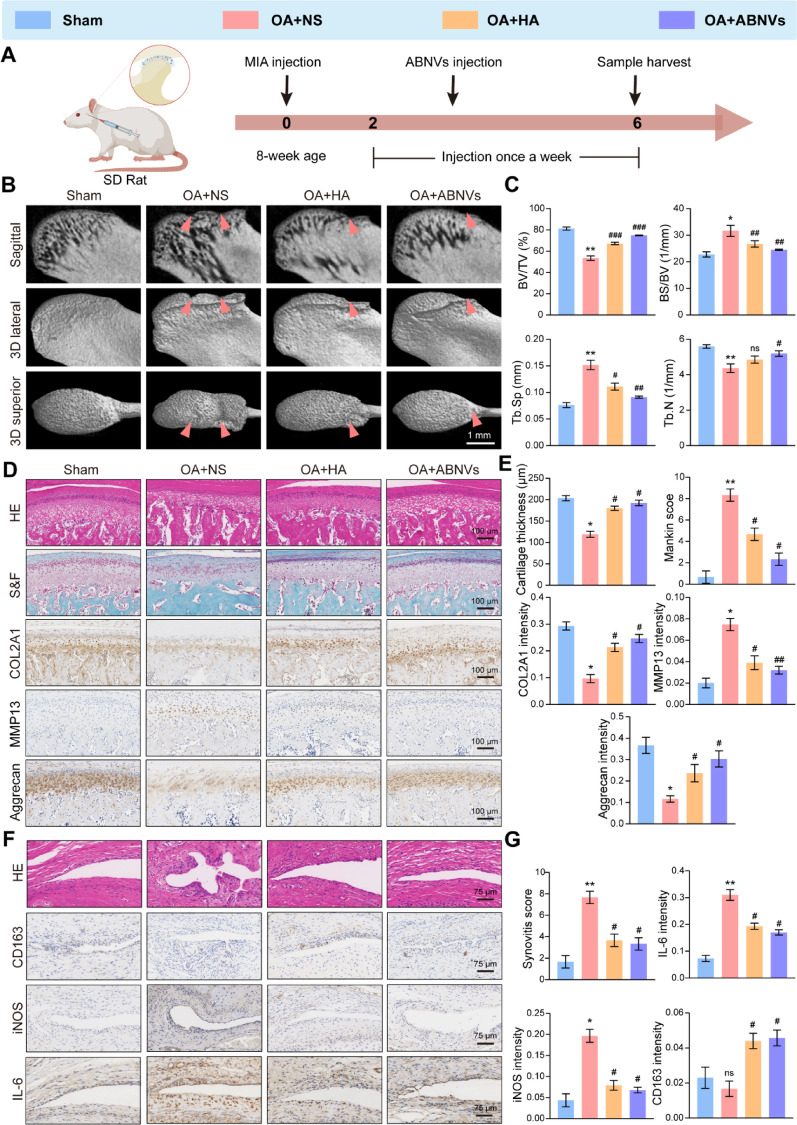
Local injection of ABNVs alleviated TMJOA in rats.** (A)** Flow chart for establishing the TMJOA model and ABNVs treatment in SD rats. **(B)** Superior and lateral views of the condylar morphology and subchondral bone changes. **(C)** Quantitative analysis of subchondral bone parameters, including BV/TV, BS/BV, Tb.N, and Tb.Sp. **(D**,** E)** Representative images of H&E staining, typical Safranin O-Fast Green, and IHC staining of COL2A1, MMP13, and Aggrecan in condylar cartilage after 4 weeks of ABNVs injection. **(F**,** G)** Representative images of H&E and IHC staining for CD163, iNOS, and IL-6 in synovial tissue after 4 weeks of ABNVs injection. Statistical analysis was conducted via one-way ANOVA (*n* = 3). Statistical significance compared with the Sham group, ^*^p < 0.05 and ^**^p < 0.01; compared with the OA + NS group, ^#^*p* < 0.05, ^##^*p* < 0.01, and ^###^*p* < 0.001. “ns” indicates no significant difference

We first explored the therapeutic effects of ABNVs in vivo by establishing a TMJOA model using monoiodoacetate (MIA) in Sprague-Dawley (SD) rats (Fig. 2A) following the standard protocol [23]. Following intra-articular injection of DiR-labeled ABNVs (ABNVs-DiR) or free DiR dye, in vivo imaging was performed on days 1, 5, and 7. By day 7, the ABNVs-DiR group still exhibited a small amount of signal (Fig. S2). This indicates that ABNVs persist in the joint cavity for over 7 days, with sustained signaling supporting our weekly dosing regimen. Three treatment groups were included: the OA + NS group (saline injection), the OA + HA group (hyaluronic acid injection), and the OA + ABNVs group (ABNVs injection).

The Sham group underwent needling without injection. HA served as a routinely used clinical therapy for TMJOA. Micro-CT scanning of condylar samples revealed that the ABNVs group exhibited improved condylar bone integrity, characterized by increased bone volume, higher trabecular number, reduced trabecular separation, and overall superior effects compared to the OA + NS group without affecting bodyweight (Fig. 2B, C, and Fig. S3).

Histological analysis using H&E staining and Safranin O-Fast Green staining showed that both HA and ABNVs’ treatment increased cartilage layer thickness, chondrocyte numbers, and proteoglycan staining intensity in the condyle (Fig. 2D, E). Compared to the OA + NS group, the ABNVs group also exhibited reduced levels of matrix metalloproteinases, such as MMP13. Notably, COL2A1 and aggrecan levels in the condylar cartilage were significantly lower in the OA + NS group compared to the Sham group, but ABNVs treatment restored COL2A1 and aggrecan levels in the TMJ (Fig. 2D, E). These findings emphasize the ability of ABNVs to restore TMJOA condylar cartilage and subchondral bone.

In the early stages of OA, macrophages accumulate in the synovial endothelium, contributing to synovial inflammation and disease progression [26]. Synovial tissue analysis revealed significant thickening of the synovial layer in the OA + NS group, along with increased inflammatory cell infiltration and blood vessel formation, indicating active synovial inflammation. In contrast, HA and ABNVs’ treatments effectively reduced synovial inflammation and synovial thickening (Fig. 2F, G). Immunohistochemical (IHC) analysis showed a significant increase in M1 macrophage markers IL-6 and iNOS expression and a decrease in M2 macrophage marker CD163 in the OA + NS group as compared to the Sham group, suggesting an increase in the activity and inflammatory intensity of M1-type macrophages. Importantly, HA or ABNVs’ treatment significantly decreased IL-6 and iNOS expression and increased CD163 expression. ABNVs decreased the number of M1 macrophages and increased the number of anti-inflammatory macrophages, demonstrating significant anti-inflammatory effects (Fig. 2F, G).

Blood samples collected from rats for liver and kidney function and routine blood analysis showed no significant changes in the individual indices, and all values remained within the normal range after treatment (Fig. S4A, B). In addition, vital organs (heart, liver, spleen, lungs, and kidneys) were collected for histopathologic analysis. H&E staining and associated scoring revealed no significant signs of inflammation or other adverse reactions (Fig. S4C, D). The results confirmed the good biosafety profile of ABNVs.

### ABNVs alleviated ECM degradation in condylar chondrocytes

**Fig. 3:**
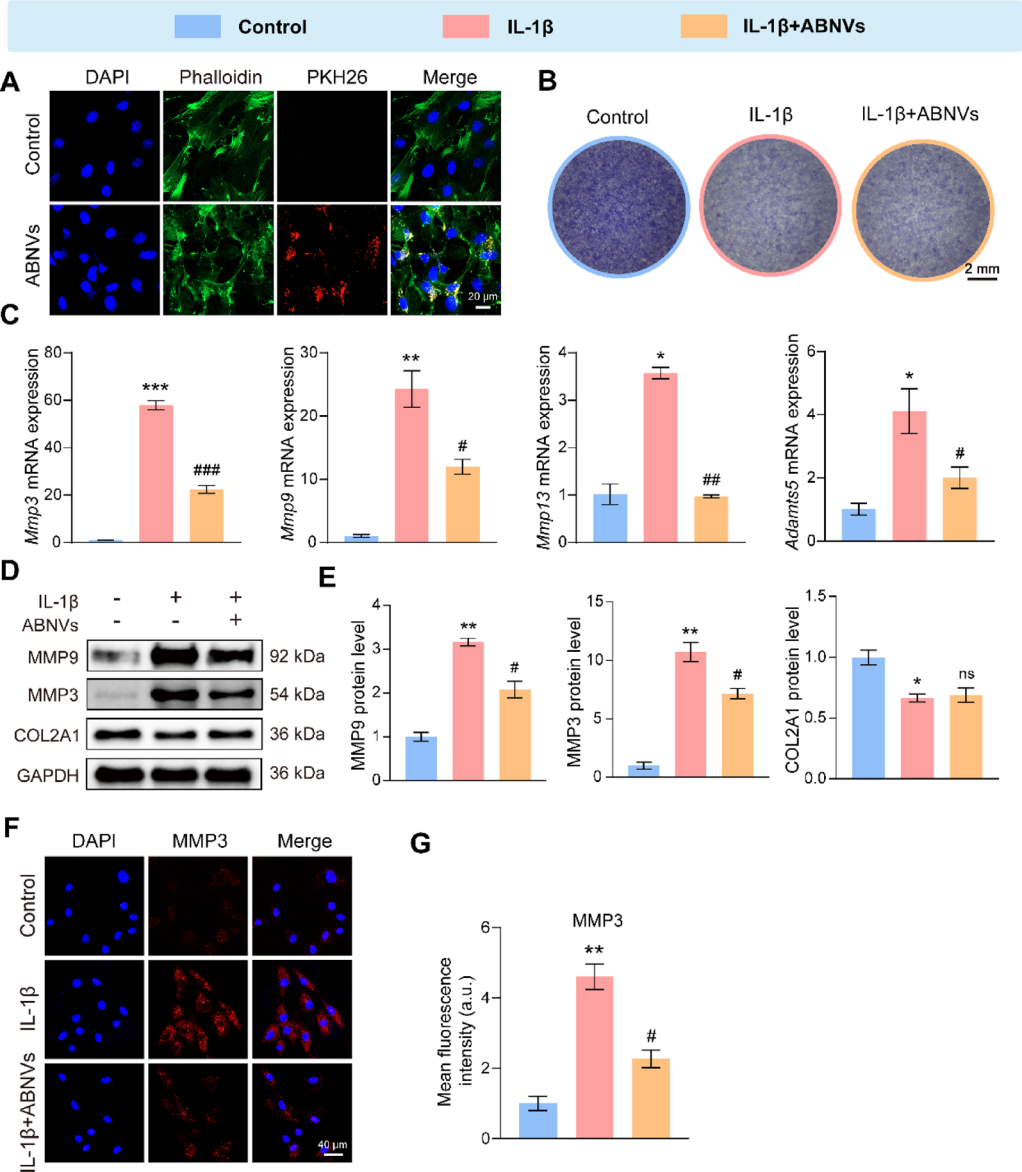
ABNVs alleviated ECM degradation in condylar chondrocytes.** (A)** Chondrocyte uptake of ABNVs (red). **(B)** Toluidine blue staining of chondrocytes cultured with ABNVs for 7 days, assessing extracellular matrix integrity. **(C)** qRT-PCR analysis of matrix degradation-related genes (*Mmp3*,* Mmp9*,* Mmp13*,* Adamts5*) in IL-1β-stimulated chondrocytes ± ABNVs. **(D**,** E)** Western blot of MMP9, MMP3, and COL2A1 in IL-1β-stimulated chondrocytes ± ABNVs. **(F**,** G)** Immunofluorescence of MMP3 (red). Data analyzed by one-way ANOVA (*n* = 3); ^*^p < 0.05, ^**^p < 0.01, ^***^p < 0.001 vs. Control; ^#^*p* < 0.05, ^##^*p* < 0.01, ^###^*p* < 0.001 vs. IL-1β group. “ns” indicates no significant difference

Cartilage is essential for condylar function, relying on the extracellular matrix (ECM) produced by chondrocytes [27]. To assess ABNVs’ role in condylar chondrocytes, we first confirmed chondrocyte identity using toluidine blue staining and COL2A1 immunofluorescence (Fig. S1A, B). PKH26-labeled ABNVs were efficiently internalized by chondrocytes (Fig. 3A), demonstrating their cellular uptake. In IL-1β-stimulated chondrocytes, ABNVs significantly reduced the expression of ECM-degrading genes (*Mmp3*,* Mmp9*,* Mmp13*,* Adamts5*; Fig. 3C) and proteins (MMP3, MMP9; Fig. 3D, E), supported by immunofluorescence (Fig. 3F, G). However, ABNVs did not restore COL2A1 levels (Fig. 3D, E) or reverse IL-1β-induced proteoglycan loss (toluidine blue staining; Fig. 3B). These findings indicate that ABNVs mitigate ECM degradation under inflammation but do not rescue ECM synthesis.

### ABNVs alleviated M1 macrophage polarization in vitro

Synovial macrophages, particularly the pro-inflammatory M1 phenotype, drive OA progression by releasing reactive oxygen species (ROS) and inflammatory cytokines (e.g., IL-1β, IL-6, TNF-α), which exacerbate ECM degradation [28, 29]. Our in vivo study showed remarkable downregulation of M1 markers and inflammatory markers in ABNVs-treated TMJ-OA tissues (Fig. 2). We further evaluated whether ABNVs could mitigate M1 macrophage polarization in vitro.

**Fig. 4:**
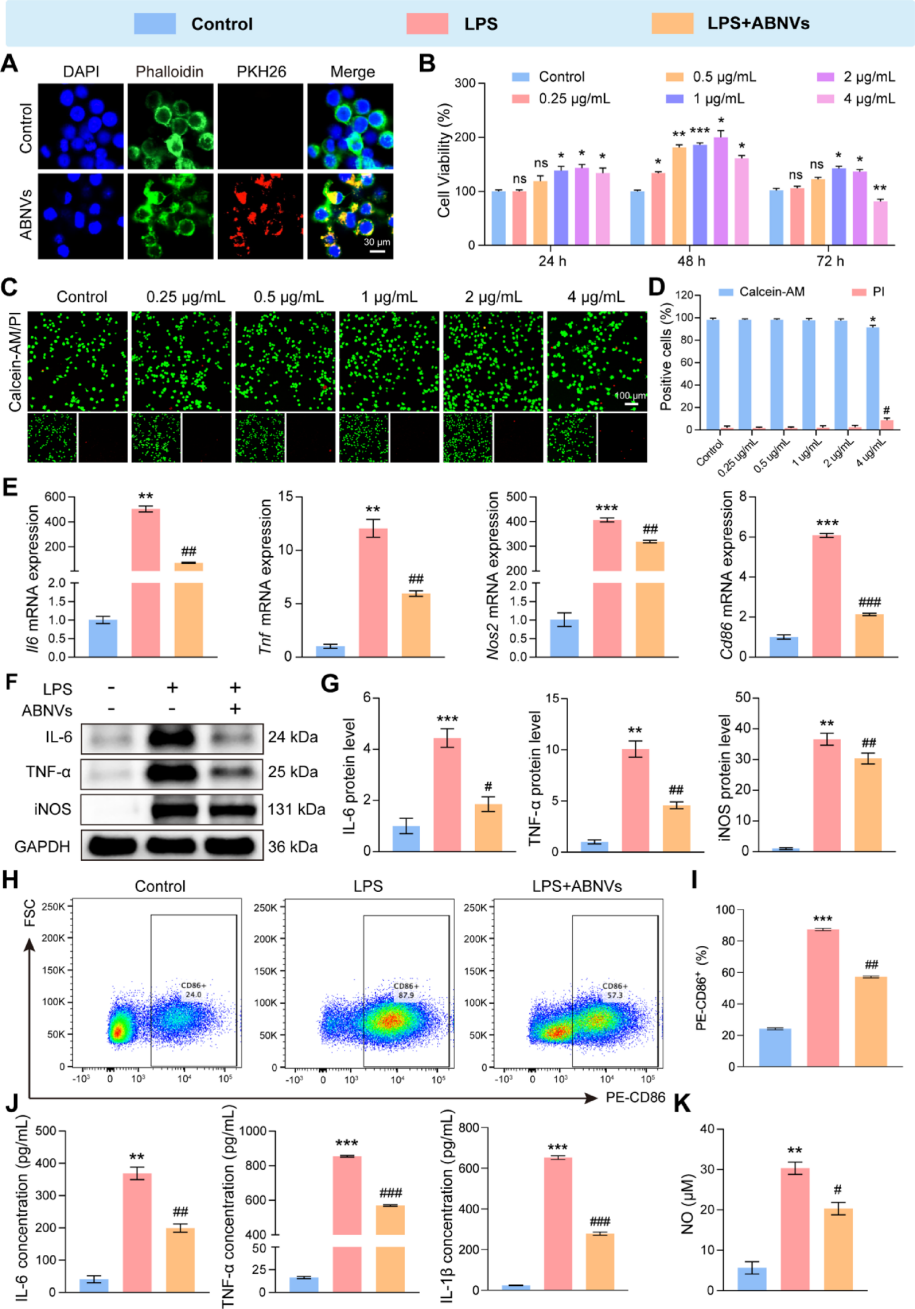
ABNVs attenuate M1 macrophage inflammation.** (A)** Macrophage uptake of PKH26-labeled ABNVs (red). **(B)** Viability of RAW264.7 macrophages treated with ABNVs (0.25–4 µg/mL) for 24–72 h (CCK-8 assay). **(C**,** D)** Live/dead staining of macrophages after ABNVs treatment. **(E)** qRT-PCR of M1 markers (*Il6*, *Tnf*, *Cd86*, *Nos2*). **(F**,** G)** Western blot of IL-6, TNF-α, and iNOS. **(H)** Representative images and **(I)** statistical results of flow cytometry detection of CD86 positivity, a marker of M1 polarization in macrophages. **(J)** ELISA of IL-6, TNF-α, and IL-1β in supernatants. **(K)** Nitric oxide (NO) levels in supernatants. Data analyzed by one-way ANOVA (*n* = 3); ^**^p < 0.01, ^***^p < 0.001 vs. Control; ^#^*p* < 0.05, ^##^*p* < 0.01, ^###^*p* < 0.001 vs. LPS group

**Fig. 5:**
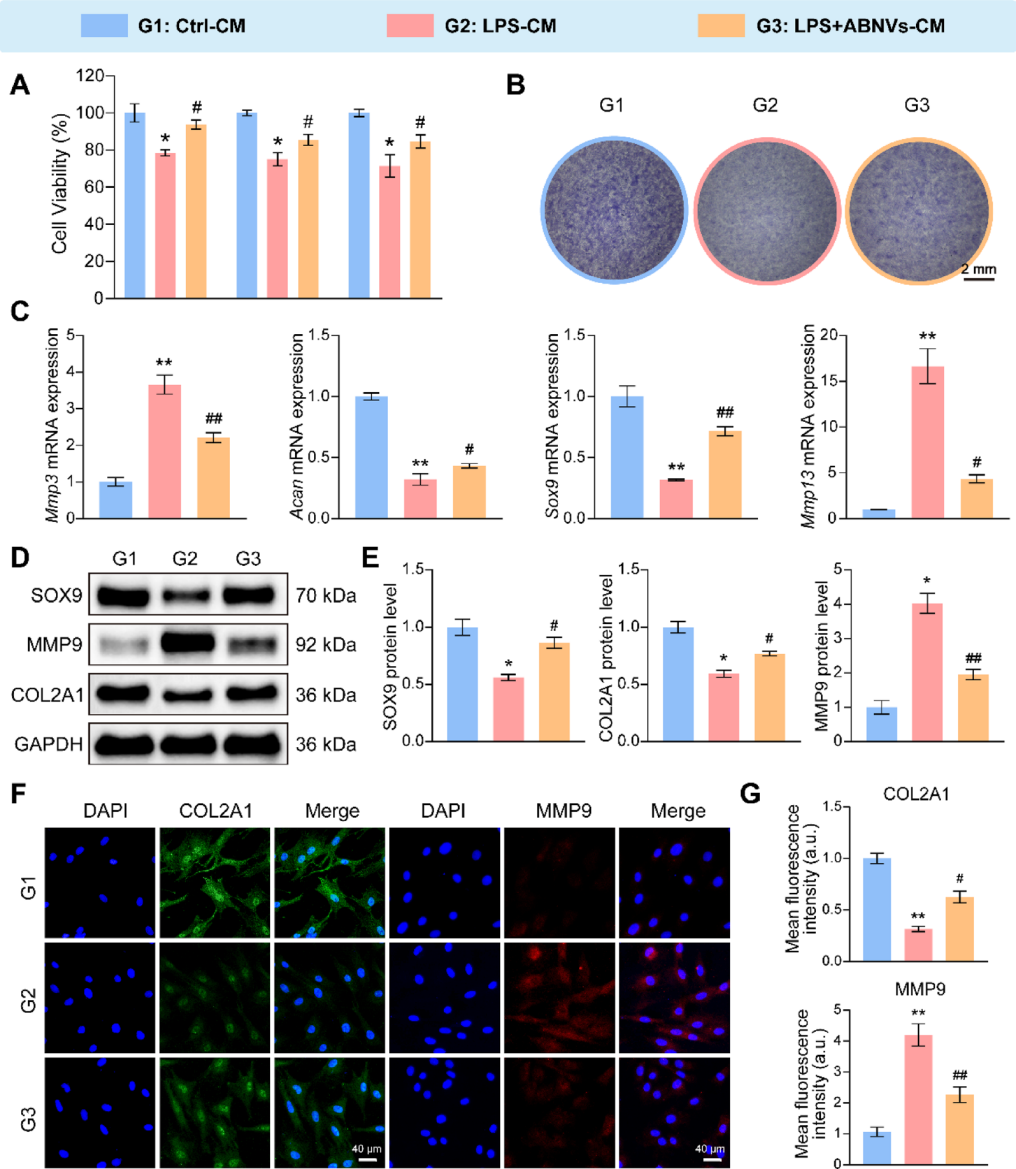
ABNVs rescue chondrocyte function by modulating macrophage responses.** (A)** Chondrocyte metabolic activity (CCK-8 assay) after 24–72 h culture with macrophage CM. **(B)** Proteoglycan content (toluidine blue staining). **(C)** RT-qPCR of matrix genes (*Mmp3*, *Mmp13*, *Acan*, *Sox9*). **(D**,** E)** Western blot and quantification of SOX9, MMP9, and COL2A1. **(F**,** G)** Immunofluorescence of COL2A1 (green) and MMP9 (red). Data analyzed by one-way ANOVA (*n* = 3); ^*^p < 0.05, ^**^p < 0.01 vs. Ctrl-CM; ^#^*p* < 0.05, ^##^*p* < 0.01 vs. LPS-CM

CCK-8 assays revealed that 4 µg/mL ABNVs inhibited macrophage survival at 72 h (Fig. 4B), corroborated by live/dead staining (Fig. 4C, D). In LPS-stimulated macrophages, 1 µg/mL ABNVs optimally suppressed inflammatory gene expression (Fig. S5) and were selected for further macrophage in vitro experiments. PKH26-labeled ABNVs were efficiently internalized by macrophages (Fig. 4A). LPS stimulation upregulated M1 markers (*Il6*,* Tnf*,* Nos2*, and *Cd86*) at mRNA (Fig. 5E and Fig. S5) and protein levels (IL-6, TNF-α, and iNOS; Fig. 4F, G). Flow cytometry demonstrated a downregulation of the surface marker CD86 on M1 macrophages following ABNVs treatment, indicating that ABNVs can suppress M1 macrophage polarization to a certain extent (Fig. 4H, I). ABNVs’ treatment significantly reduced these inflammatory mediators. Additionally, ABNVs decreased secreted IL-6, TNF-α, IL-1β, and nitric oxide (NO) in culture supernatants (Fig. 4J, K), confirming their anti-inflammatory efficacy in macrophages.

### ABNVs restored chondrocyte functions in vitro via modulation of the macrophage inflammatory response

Our investigation of macrophage-chondrocyte crosstalk in TMJOA used conditioned media (CM) from: (1) control macrophages (Ctrl-CM), (2) LPS-induced M1 macrophages (LPS-CM), and (3) ABNVs-treated M1 macrophages (LPS + ABNVs-CM) [30]. LPS-CM significantly reduced chondrocyte viability and migration compared to Ctrl-CM (Fig. 5A and Fig. S6A, B), while LPS + ABNVs-CM attenuated these effects. Toluidine blue staining revealed LPS + ABNVs-CM prevented LPS-CM-induced proteoglycan loss (Fig. 5B). At the molecular level, LPS-CM upregulated matrix-degrading genes (*Mmp3*, *Mmp13*) and downregulated matrix-forming genes (*Sox9*, *Acan*), with LPS + ABNVs-CM partially reversing these changes (Fig. 5C). Western blot analysis confirmed ABNVs reduced LPS-CM-induced MMP9 overexpression and restored COL2A1/SOX9 levels (Fig. 5D, E), supported by immunofluorescence (Fig. 5F, G). To further validate the paracrine effects of macrophages, we also co-cultured macrophages and chondrocytes using the Transwell system. Specifically, untreated (control) and LPS-induced M1-type RAW264.7 cells were placed in the upper chamber. Similarly, ABNVs were added to the upper chamber, while condylar chondrocytes were placed in the bottom wells. The results closely mirrored those obtained with conditioned medium: ABNVs treatment alleviated M1 macrophage-induced degradation of the cartilage matrix and restored levels of COL2A1 and proteoglycans (Fig. S7A-D). These findings demonstrate ABNVs counteract M1 macrophage-mediated chondrocyte dysfunction in TMJOA by modulating inflammatory responses, suggesting therapeutic potential for cartilage protection.

### CAT- and SOD-enriched ABNVs reduced oxidative stress in M1 macrophages

**Fig. 6:**
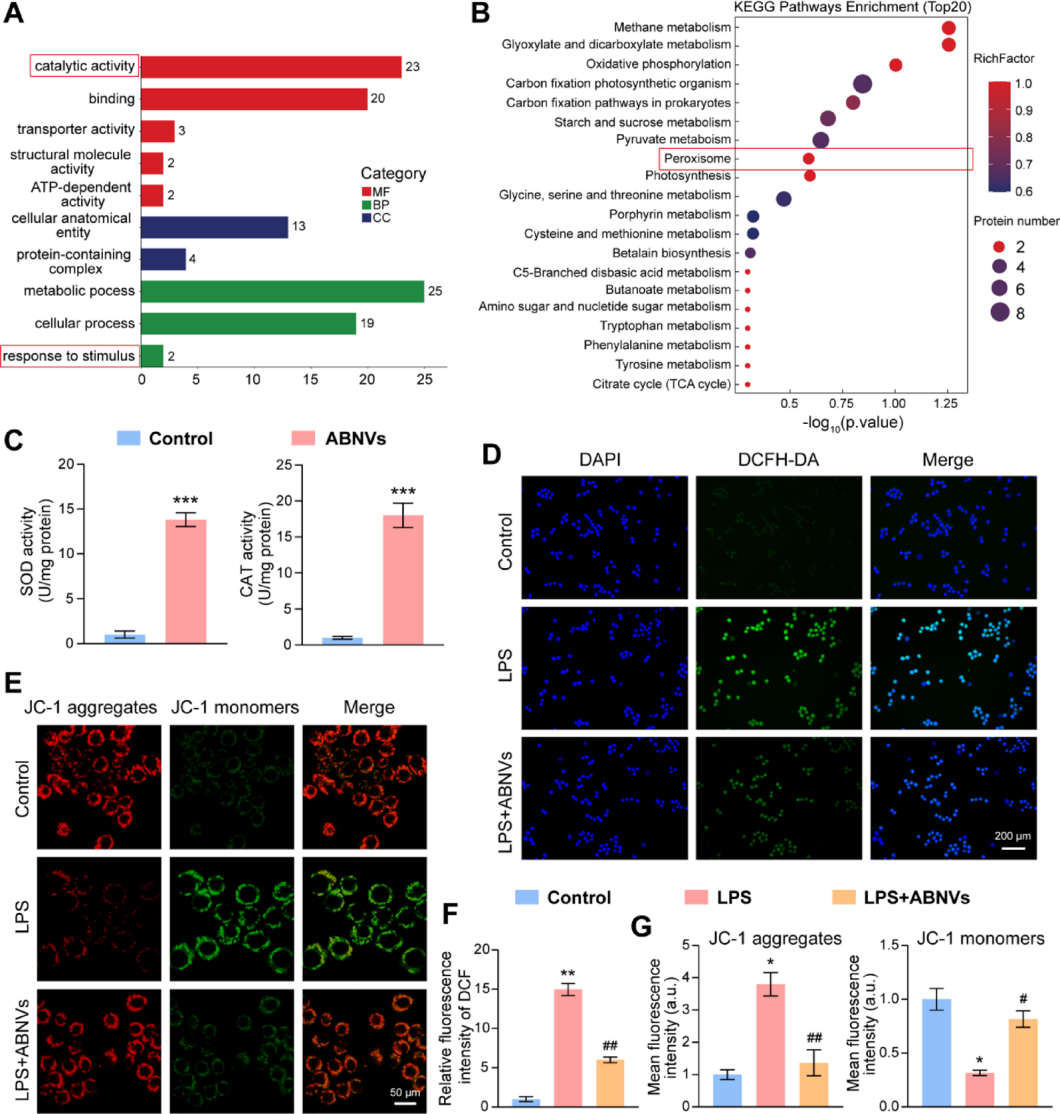
Antioxidant effects of CAT- and SOD-enriched ABNVs in macrophages.** (A**,** B)** GO and KEGG enrichment analysis of top 50 ABNVs proteins. **(C)** CAT and SOD enzymatic activity in ABNVs. **(D**,** F)** Intracellular ROS levels (DCFH-DA, green) and quantification. **(E**,** G)** Mitochondrial membrane potential (JC-1 staining; red: high ΔΨm, green: low ΔΨm). Data analyzed by Student’s t-test and one-way ANOVA (*n* = 3); ^*^p < 0.05, ^**^p < 0.01, ^***^p < 0.001 vs. Control; ^#^*p* < 0.05, ^##^*p* < 0.01 vs. LPS group

Our proteomic analysis of ABNVs identified catalase (CAT) and superoxide dismutase (SOD) as key antioxidant components, with KEGG and GO enrichment analyses highlighting their roles in catalytic activity and stress response among the top 50 proteins (Fig. 6A, B). CAT and SOD accounted for 2.21% and 0.18% of ABNVs proteins, respectively, with the majority of the remaining proteins being plant-specific or structural in function (Fig. S8). Functional assays confirmed these enzymes maintain activity within ABNVs (Fig. 6C), supporting their potential to combat oxidative stress.

ABNVs exhibited a distinct enzyme profile compared to root extracts, with higher CAT but slightly lower SOD activity, suggesting the vesicle interior is better suited for CAT (Fig. S9A). Trypsin treatment confirmed that both enzymes are encapsulated within the lipid bilayer, protecting them from degradation (Fig. S9B). Furthermore, ABNVs demonstrated remarkable stability after lyophilization and one month of storage at −80 °C. They maintained their cup-shaped structure, particle size, and dispersibility without aggregation (Fig. S10A, C). While the zeta potential slightly decreased, it remained stable, and enzyme activity showed only a minimal, non-progressive loss (Fig. S10B, D, E). In conclusion, freeze-drying is an effective method for preserving ABNVs structure and bioactivity for short-term storage.

Since CAT and SOD work synergistically to neutralize ROS, with SOD converting superoxide to hydrogen peroxide, which CAT then breaks down to water and oxygen [31], we evaluated their therapeutic effects in OA. In LPS-stimulated macrophages, ABNVs significantly reduced intracellular ROS levels (Fig. 6D, F) and restored mitochondrial membrane potential (ΔΨm), as evidenced by increased JC-1 aggregate (red) fluorescence and decreased monomeric (green) fluorescence (Fig. 6E, G). Parallel antioxidant effects were observed in chondrocytes (Fig. S11A, B), demonstrating broad cellular protection. These findings establish that CAT- and SOD-enriched ABNVs mitigate oxidative damage by both scavenging ROS and preserving mitochondrial function [32], offering a promising approach to disrupt ROS-mediated inflammation in OA.

### ABNVs inhibit JNK/FOXO1 signaling to attenuate macrophage inflammation

**Fig. 7:**
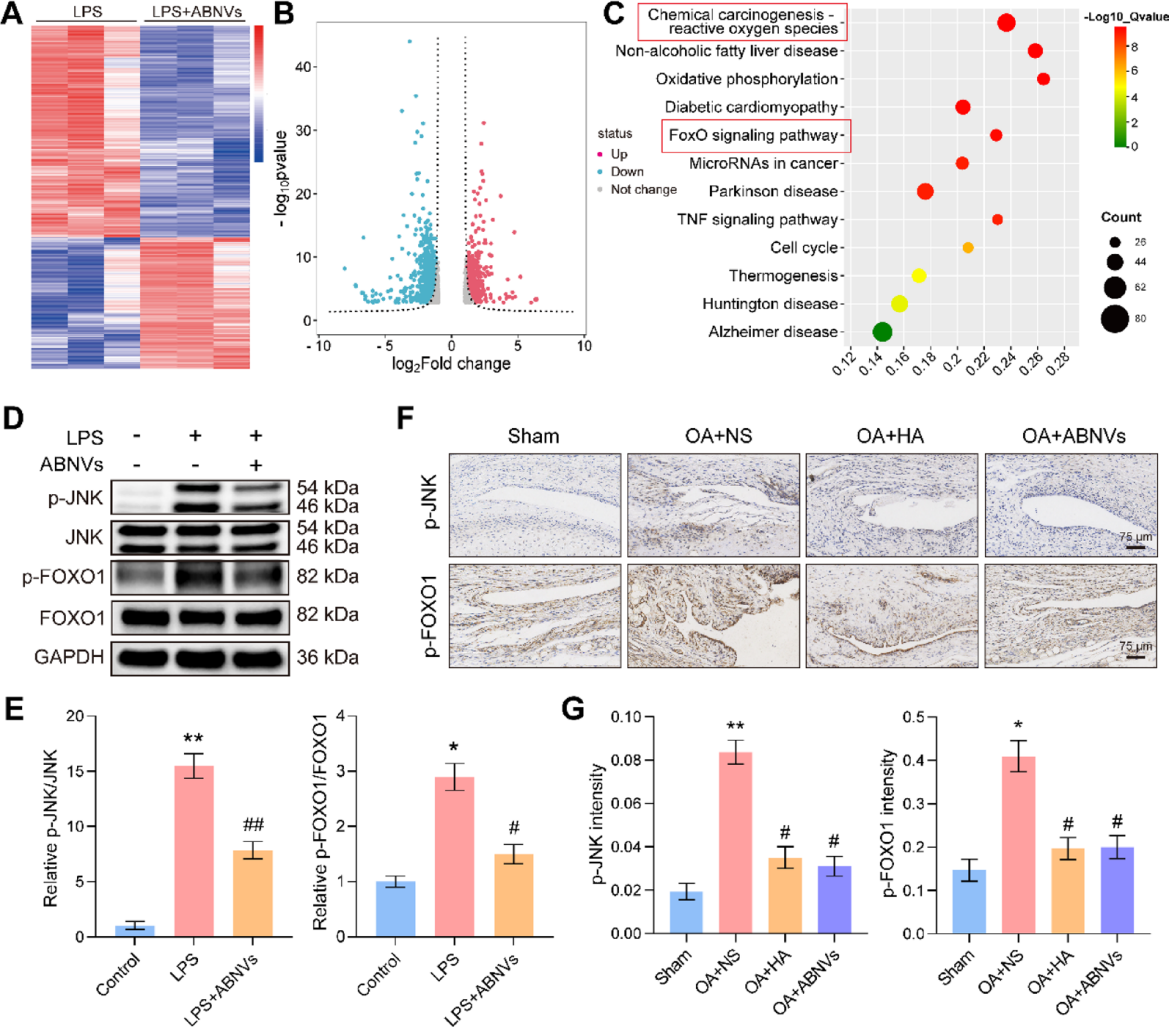
ABNVs inhibit JNK/FOXO1 signaling in M1 macrophages.** (A)** Heatmap and **(B)** volcano plot of differentially expressed genes (DEGs) in LPS ± ABNVs-treated macrophages. **(C)** KEGG pathway enrichment of DEGs. **(D**,** E)** Western blot and quantification of p-JNK/JNK and p-FOXO1/FOXO1. **(F**,** G)** IHC staining and quantification of p-JNK and p-FOXO1 in TMJ synovial tissue. Data analyzed by one-way ANOVA (*n* = 3); ^*^p < 0.05, ^**^p < 0.01 vs. Control/Sham; ^#^*p* < 0.05, ^##^*p* < 0.01 vs. LPS/OA + NS

To elucidate the anti-inflammatory mechanism of ABNVs in macrophages, we performed transcriptome sequencing of LPS-stimulated M1 macrophages treated with ABNVs, identifying 1,652 differentially expressed genes (632 upregulated, 1,020 downregulated) (Fig. 7A, B). KEGG pathway analysis revealed significant alterations in the chemical carcinogenesis-reactive oxygen species pathway (Fig. 7C), which is closely associated with MAPK and FOXO signaling pathways [33]. Given that FOXO1, a key regulator of macrophage polarization, is activated by JNK (a MAPK family member) in response to ROS [34, 35], we hypothesized that ABNVs attenuate inflammation through ROS-mediated JNK/FOXO1 inhibition. Western blot analysis confirmed ABNVs significantly reduced phosphorylation of both JNK and FOXO1 in RAW264.7 macrophages (Fig. 7D, E), with immunohistochemical staining of rat synovial tissue showing consistent suppression of p-JNK and p-FOXO1 (Fig. 7F, G). These findings demonstrate that ABNVs exert their anti-inflammatory effects by disrupting the ROS-JNK-FOXO1 signaling axis, thereby inhibiting M1 macrophage polarization.

### Mechanistic validation of abnvs’ anti-inflammatory effects through CAT/SOD-JNK/FOXO1 axis

**Fig. 8:**
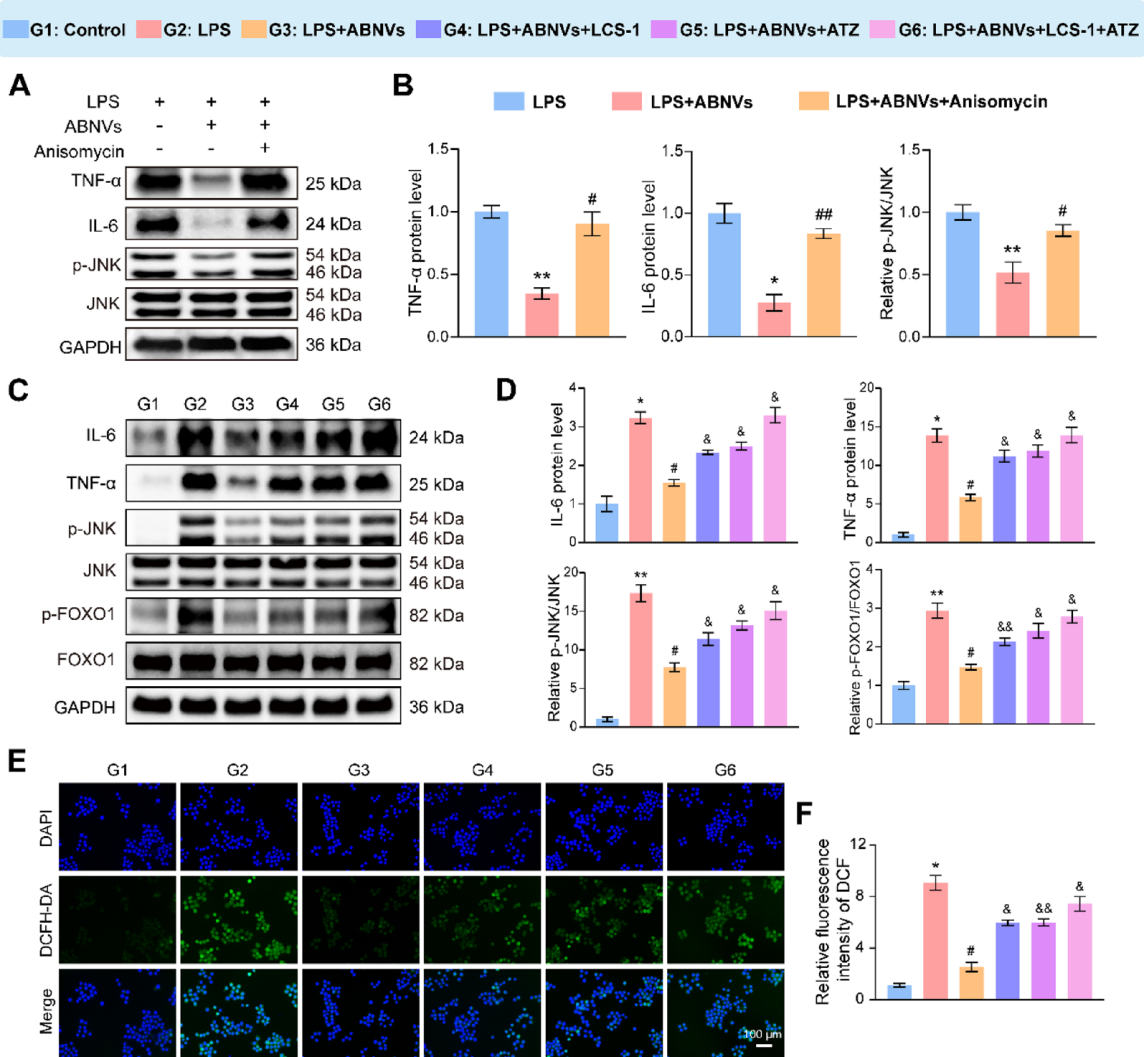
ABNVs reduce inflammation through ROS scavenging and JNK/FOXO1 inhibition.** (A**,** B)** Western blot analysis of IL-6, TNF-α, and p-JNK/JNK following anisomycin treatment. **(C**,** D)** Western blot of IL-6, TNF-α, p-JNK/JNK, and p-FOXO1/FOXO1 after LCS-1 (SOD inhibitor) and ATZ (CAT inhibitor) treatment. **(E**,** F)** Intracellular ROS levels (DCFH-DA staining, green) and quantification. Data analyzed by one-way ANOVA (*n* = 3); ^*^p < 0.05, ^**^p < 0.01 vs. Control; ^#^*p* < 0.05, ^##^*p* < 0.01 vs. LPS; ^&^*p* < 0.05, ^&&^*p* < 0.01 vs. LPS + ABNVs

Our mechanistic studies demonstrate that CAT- and SOD-enriched ABNVs alleviate inflammation through ROS scavenging and JNK/FOXO1 pathway modulation. To directly validate this pathway, we employed the JNK activator anisomycin (25 ng/mL) [34], which reversed ABNVs’ suppression of p-JNK phosphorylation and restored IL-6/TNF-α production (Fig. 8A, B), confirming JNK/FOXO1’s essential role. CCK8 results indicate that the use of anisomycin has no significant effect on cell viability (Fig. S12). Building on our identification of functional CAT and SOD in ABNVs (Fig. 6C), we directly applied exogenous CAT and SOD to M1 macrophages. The results demonstrated that both enzymes exhibit potent anti-inflammatory effects while simultaneously inhibiting JNK activation (Fig. S13A, B). Additionally, we used specific enzyme inhibitors (ATZ [36] for CAT; LCS-1 [37] for SOD) to probe their contribution. ABNVs were incubated with each inhibitor at 37℃ for 3 h. Enzyme assays revealed that inhibitor-treated ABNVs exhibited a statistically significant reduction in corresponding enzyme activity, while the control group maintained baseline activity (Fig. S13C). This confirms that both inhibitors selectively and effectively block their target enzymes in ABNVs. Notably, inhibitor treatment abolished ABNVs’ anti-inflammatory effects, increasing: (1) IL-6/TNF-α levels, (2) JNK/FOXO1 phosphorylation (Fig. 8C, D), and (3) intracellular ROS accumulation (Fig. 8E, F).

Macrophages were treated with 100 µM H₂O₂ to induce oxidative stress [38], under the following conditions: (1) control, (2) H₂O₂, (3) H₂O₂ + ABNVs, and (4) H₂O₂ + ABNVs + LCS‑1 + ATZ. Results showed that H₂O₂ significantly increased ROS (Fig. S14A, B) and p‑JNK levels (Fig. S14C, D), and these effects were effectively reversed by ABNVs. The co-administration of ABNVs with enzyme inhibitors significantly attenuated their protective effects, as evidenced by the return of ROS levels and p-JNK activation to near-baseline (model) conditions. This result establishes a direct causal pathway: ABNVs suppress JNK activation by scavenging ROS, and this mechanism is negated when their ROS-clearing capacity is inhibited. Together, these gain and loss-of-function experiments establish a clear mechanistic cascade: ABNVs’ CAT/SOD enzymes reduce ROS, leading to JNK/FOXO1 pathway inhibition and subsequent inflammation suppression in macrophages.

## Discussion

TMJOA is a debilitating condition characterized by progressive cartilage degradation, subchondral bone remodeling, and chronic pain, significantly impairing jaw function and quality of life [39]. Current treatments, including conservative approaches (occlusal splints, intra-articular injections) and surgical interventions, offer limited disease-modifying effects or carry substantial risks [5–7]. This unmet clinical need has driven interest in plant-derived exosome-like nanovesicles (PENs), which combine the therapeutic potential of natural biomolecules with the advantages of scalable production and biocompatibility [40–42]. Among these, *Achyranthes bidentata*-derived nanovesicles (ABNVs) emerge as a promising candidate, addressing both the mechanistic drivers of TMJOA and the practical challenges of clinical translation.

Our study demonstrates that ABNVs, enriched with catalase (CAT) and superoxide dismutase (SOD), exert dual therapeutic effects: (1) direct ROS scavenging and (2) suppression of macrophage-mediated inflammation via JNK/FOXO1 pathway inhibition. These findings bridge the gap between traditional medicine and nanotechnology, as *Achyranthes bidentata* has long been recognized for its anti-inflammatory and chondroprotective properties [12]. However, crude extracts suffer from poor bioavailability and instability, limitations overcome by ABNVs through their natural encapsulation of bioactive components. In vivo, ABNVs outperformed hyaluronic acid, the current clinical standard, by mitigating cartilage degradation and synovial inflammation in a rat TMJOA model, while exhibiting no systemic toxicity.

The ROS-inflammation axis is central to OA pathogenesis, with excessive ROS driving mitochondrial dysfunction, cartilage breakdown, and sustained inflammation [43, 44]. ABNVs disrupt this cycle through their CAT/SOD cargo, which scavenges ROS and subsequently inhibits ROS-activated JNK signaling.

ABNVs present a significant advantage over synthetic nanoformulations by embodying a naturally optimized, “ready-to-use” nanoplatform. Although sophisticated synthetic nanoreactors co-delivering CAT and SOD have been engineered for enhanced cascade reactions in anti-inflammatory therapy [8], their development faces inherent hurdles in complexity, immunogenicity, and scalability. ABNVs, in contrast, inherently possess a biocompatible lipid bilayer that natively and efficiently co-encapsulates these enzymes in their active conformations. This evolutionarily refined architecture not only ensures targeted delivery but also circumvents the considerable challenges of synthetic design and manufacturing. ABNVs reduce pro-inflammatory cytokine secretion (e.g., TNF-α, IL-6) and prevent M1 macrophage polarization [35, 45], as evidenced by decreased p-JNK/p-FOXO1 levels and restored chondrocyte matrix integrity. Notably, the ABNVs-treated macrophages reversed the catabolic effects of M1 macrophages on chondrocytes, underscoring the therapeutic potential of targeting macrophage-chondrocyte crosstalk [30, 46, 47]. Beyond TMJOA, ABNVs exemplify the broader promise of PENs, which offer targeted delivery, stability, and diverse administration routes [48–52]. For instance, ginseng-derived exosomes promote wound healing [49], while *Pueraria lobata* exosomes ameliorate osteoporosis [50] and colitis [51, 52]. Our work extends these applications to joint disorders, highlighting ABNVs’ potential for inflammatory and age-related conditions.

While this study highlights the therapeutic promise of ABNVs for TMJOA, some experimental limitations should be noted. First, the in vivo validation was performed in an acute MIA-induced rat model, which may not fully replicate the chronic, low-grade inflammation characteristic of human TMJOA progression. Second, the therapeutic concentration of ABNVs was optimized in vitro (1 µg/mL) but requires further pharmacokinetic studies to determine optimal dosing regimens for clinical translation. Future work should incorporate chronic disease models and advanced co-culture systems to better understand the temporal and spatial dynamics of ABNVs-mediated joint protection.

## Conclusion

In summary, ABNVs represent a breakthrough in TMJOA therapy, combining the therapeutic heritage of *Achyranthes bidentata* with the advantages of nanovesicle technology. By delivering functional CAT and SOD, ABNVs directly scavenge ROS and suppress JNK/FOXO1-mediated inflammation, effectively mitigating cartilage degradation and synovitis in vivo. Their superior efficacy over hyaluronic acid, coupled with excellent biosafety, positions ABNVs as a clinically translatable candidate for TMJOA and potentially other inflammatory joint disorders. Future research should focus on optimizing delivery strategies and evaluating long-term therapeutic outcomes to realize the full potential of this innovative platform.

## Supplementary Information

Supplementary Material 1

## Data Availability

Data will be made available on request.

## Abbreviations

TMJOA: Temporomandibular joint osteoarthritis
ABNVs: Achyranthes bidentata-derived exosome-like nanovesicles
MPENs: Medicinal plant exosome-like nanovesicles
CAT: Catalase
SOD: Superoxide dismutase
ROS: Reactive oxygen species
NO: Nitric oxide
ECM: Extracellular matrix
OA: Osteoarthritis
FBS: Fetal bovine serum
H&E: Hematoxylin and eosin
S&F: Safranin-O/fast green
LPS: Lipopolysaccharide
CM: Conditioned medium
NS: Normal saline
BV/TV: The ratio of bone volume to tissue volume
BS/BV: The ratio of bone surface to bone volume
Tb.N: Trabecular number
Tb.Sp: Trabecular space
MIA: Monoiodoacetate

## References

[1] Wan J, Lin J, Zha T, Ciruela F, Jiang S, Wu Z, et al. Temporomandibular disorders and mental health: shared etiologies and treatment approaches. J Headache Pain. 2025;26(1):52. 10.1186/s10194-025-01985-6. 40075300 10.1186/s10194-025-01985-6 PMC11899861

[2] Lu K, Ma F, Yi D, Yu H, Tong L, Chen D. Molecular signaling in temporomandibular joint osteoarthritis. J Orthop Transl. 2022;32:21–7. 10.1016/j.jot.2021.07.001. 10.1016/j.jot.2021.07.001 PMC9072795 35591935

[3] Acri TM, Shin K, Seol D, Laird NZ, Song I, Geary SM, et al. Tissue engineering for the temporomandibular joint. Adv Healthc Mater. 2019;8(2):e1801236. 10.1002/adhm.201801236. 30556348 10.1002/adhm.201801236 PMC7075314

[4] Zhou J, Ren R, Li Z, Zhu S, Jiang N. Temporomandibular joint osteoarthritis: a review of animal models induced by surgical interventions. Oral Dis. 2023;29(7):2521–8. 10.1111/odi.14266. 35615772 10.1111/odi.14266

[5] Huang X, Pan X, Xiong X, Zhao Z, Cen X. Drug delivery systems for treatment of temporomandibular joint osteoarthritis. Front Pharmacol. 2022;13:1054703. 10.3389/fphar.2022.1054703. 36419625 10.3389/fphar.2022.1054703 PMC9676453

[6] Wang C, Wang Y, Wang C, Shi J, Wang H. Research progress on tissue engineering in repairing temporo-mandibular joint. Zhejiang Da Xue Xue Bao Yi Xue Ban. 2021;50(2):212–21. 10.3724/zdxbyxb-2021-0118. 34137227 10.3724/zdxbyxb-2021-0118 PMC8710277

[7] Sah MK, Abdelrehem A, Chen S, Shen P, Jiao Z, Hu YK, et al. Prognostic indicators of arthroscopic discopexy for management of temporomandibular joint closed lock. Sci Rep. 2022;12(1):3194. 10.1038/s41598-022-07014-9. 35210483 10.1038/s41598-022-07014-9 PMC8873273

[8] Kwon K, Jung J, Sahu A, Tae G. Nanoreactor for cascade reaction between SOD and CAT and its tissue regeneration effect. J Control Release. 2022;344:160–72. 10.1016/j.jconrel.2022.02.033. 35247490 10.1016/j.jconrel.2022.02.033

[9] Liu X, Li Y, Zhao J, Hu Z, Fang W, Ke J, et al. Pyroptosis of chondrocytes activated by synovial inflammation accelerates TMJ osteoarthritis cartilage degeneration via ROS/NLRP3 signaling. Int Immunopharmacol. 2023;124:110781. 10.1016/j.intimp.2023.110781. 37625369 10.1016/j.intimp.2023.110781

[10] Zhang H, Cai D, Bai X. Macrophages regulate the progression of osteoarthritis. Osteoarthritis Cartilage. 2020;28(5):555–61. 10.1016/j.joca.2020.01.007. 31982565 10.1016/j.joca.2020.01.007

[11] Manferdini C, Paolella F, Gabusi E, Silvestri Y, Gambari L, Cattini L, et al. From osteoarthritic synovium to synovial-derived cells characterization: synovial macrophages are key effector cells. Arthritis Res Ther. 2016;18:83. 10.1186/s13075-016-0983-4. 27044395 10.1186/s13075-016-0983-4 PMC4820904

[12] Weng X, Lin P, Liu F, Chen J, Li H, Huang L, et al. *Achyranthes bidentata* polysaccharides activate the Wnt/β-catenin signaling pathway to promote chondrocyte proliferation. Int J Mol Med. 2014;34(4):1045–50. 10.3892/ijmm.2014.1869. 25176272 10.3892/ijmm.2014.1869

[13] Xie W, Qi S, Dou L, Wang L, Wang X, Bi R, et al. Achyranthoside D attenuates chondrocyte loss and inflammation in osteoarthritis via targeted regulation of Wnt3a. Phytomedicine. 2023;111:154663. 10.1016/j.phymed.2023.154663. 36657317 10.1016/j.phymed.2023.154663

[14] Li M, Zhu Y, Peng W, Wang H, Yuan Y, Gu X. *Achyranthes bidentata* polypeptide protects Schwann cells from apoptosis in hydrogen peroxide-induced oxidative stress. Front Neurosci. 2018;12:868. 10.3389/fnins.2018.00868. 30555292 10.3389/fnins.2018.00868 PMC6284036

[15] Aqil F, Munagala R, Jeyabalan J, Agrawal AK, Gupta R. Exosomes for the enhanced tissue bioavailability and efficacy of curcumin. AAPS J. 2017;19(6):1691–702. 10.1208/s12248-017-0154-9. 29047044 10.1208/s12248-017-0154-9

[16] Fu J, Liu Z, Feng Z, Huang J, Shi J, Wang K, et al. *Platycodon grandiflorum* exosome-like nanoparticles: the material basis of fresh *Platycodon grandiflorum* optimality and its mechanism in regulating acute lung injury. J Nanobiotechnol. 2025;23(1):270. 10.1186/s12951-025-03331-z. 10.1186/s12951-025-03331-z PMC11969861 40186259

[17] Wei Y, Cai X, Wu Q, Liao H, Liang S, Fu H, et al. Extraction, isolation, and component analysis of Turmeric-derived exosome-like nanoparticles. Bioengineering. 2023. 10.3390/bioengineering10101199. 37892929 10.3390/bioengineering10101199 PMC10604281

[18] Qiu B, Xu X, Yi P, Hao Y. Curcumin reinforces MSC-derived exosomes in attenuating osteoarthritis via modulating the miR-124/NF-kB and miR-143/ROCK1/TLR9 signalling pathways. J Cell Mol Med. 2020;24(18):10855–65. 10.1111/jcmm.15714. 32776418 10.1111/jcmm.15714 PMC7521270

[19] Yıldırım M, Ünsal N, Kabataş B, Eren O, Şahin F. Effect of *solanum lycopersicum* and *citrus limon*-derived exosome-like vesicles on chondrogenic differentiation of adipose-derived stem cells. Appl Biochem Biotechnol. 2024;196(1):203–19. 10.1007/s12010-023-04491-0. 37103740 10.1007/s12010-023-04491-0

[20] Chen P, Liu X, Gu C, Zhong P, Song N, Li M, et al. A plant-derived natural photosynthetic system for improving cell anabolism. Nature. 2022;612(7940):546–54. 10.1038/s41586-022-05499-y. 36477541 10.1038/s41586-022-05499-y PMC9750875

[21] Dad HA, Gu T-W, Zhu A-Q, Huang L-Q, Peng L-H. Plant exosome-like nanovesicles: emerging therapeutics and drug delivery nanoplatforms. Mol Ther. 2021;29(1):13–31. 10.1016/j.ymthe.2020.11.030. 33278566 10.1016/j.ymthe.2020.11.030 PMC7791080

[22] Wu A, Pathak JL, Li X, Cao W, Zhong W, Zhu M, et al. Human salivary Histatin-1 attenuates osteoarthritis through promoting M1/M2 macrophage transition. Pharmaceutics. 2023. 10.3390/pharmaceutics15041272. 37111757 10.3390/pharmaceutics15041272 PMC10147060

[23] Hei Y, Du J, Deng Z, Deng Y, Guan Y, Yang J, et al. Therapeutic effects of PEG-modified polyamide amine dendrimer for cell free DNA adsorption in temporomandibular joint osteoarthritis. ACS Appl Mater Interfaces. 2024;16(30):39153–64. 10.1021/acsami.4c08569. 39018481 10.1021/acsami.4c08569

[24] Zhong W, Li X, Pathak JL, Chen L, Cao W, Zhu M, et al. Dicalcium silicate microparticles modulate the differential expression of circRNAs and mRNAs in BMSCs and promote osteogenesis via circ_1983-miR-6931-Gas7 interaction. Biomater Sci. 2020;8(13):3664–77. 10.1039/d0bm00459f. 32463418 10.1039/d0bm00459f

[25] Chen BY, Pathak JL, Lin HY, Guo WQ, Chen WJ, Luo G, et al. Inflammation triggers chondrocyte ferroptosis in TMJOA via HIF-1α/TFRC. J Dent Res. 2024;103(7):712–22. 10.1177/00220345241242389. 38766865 10.1177/00220345241242389

[26] Sun AR, Friis T, Sekar S, Crawford R, Xiao Y, Prasadam I. Is synovial macrophage activation the inflammatory link between obesity and osteoarthritis? Curr Rheumatol Rep. 2016;18(9):57. 10.1007/s11926-016-0605-9. 27422277 10.1007/s11926-016-0605-9

[27] Li B, Guan G, Mei L, Jiao K, Li H. Pathological mechanism of chondrocytes and the surrounding environment during osteoarthritis of temporomandibular joint. J Cell Mol Med. 2021;25(11):4902–11. 10.1111/jcmm.16514. 33949768 10.1111/jcmm.16514 PMC8178251

[28] Shapouri-Moghaddam A, Mohammadian S, Vazini H, Taghadosi M, Esmaeili S-A, Mardani F, Seifi B, Mohammadi A, Afshari JT, Sahebkar A. Macrophage plasticity, polarization, and function in health and disease. J Cell Physiol. 2018;233(9):6425–40. 10.1002/jcp.26429. 29319160 10.1002/jcp.26429 PMC13482560

[29] Li H, Yuan Y, Zhang L, Xu C, Xu H, Chen Z. Reprogramming macrophage Polarization, depleting ROS by Astaxanthin and Thioketal-Containing polymers delivering Rapamycin for osteoarthritis treatment. Adv Sci (Weinh). 2024;11(9):e2305363. 10.1002/advs.202305363. 38093659 10.1002/advs.202305363 PMC10916582

[30] Jin Y, Zhang Q, Qin X, Liu Z, Li Z, Zhong X, et al. Carbon dots derived from folic acid attenuates osteoarthritis by protecting chondrocytes through NF-κB/MAPK pathway and reprogramming macrophages. J Nanobiotechnol. 2022;20(1):469. 10.1186/s12951-022-01681-6. 10.1186/s12951-022-01681-6 PMC9632154 36329497

[31] Klotz L-O, Sánchez-Ramos C, Prieto-Arroyo I, Urbánek P, Steinbrenner H, Monsalve M. Redox regulation of FoxO transcription factors. Redox Biol. 2015;6:51–72. 10.1016/j.redox.2015.06.019. 26184557 10.1016/j.redox.2015.06.019 PMC4511623

[32] Sun H, Zhan M, Zou Y, Ma J, Liang J, Tang G, et al. Bioactive phosphorus dendrimers deliver protein/drug to tackle osteoarthritis via cooperative macrophage reprogramming. Biomaterials. 2025;316:122999. 10.1016/j.biomaterials.2024.122999. 39647219 10.1016/j.biomaterials.2024.122999

[33] Wang C, Zhao X, Jiang J, Jia M, Shi W, Wu Z, et al. Integrated chemical analysis, metabolic profiling, network pharmacology, molecular docking and toxicity prediction to reveal the active ingredients and their safety of raw and prepared rhubarbs in the treatment of gastric ulcers. Front Pharmacol. 2024;15:1481091. 10.3389/fphar.2024.1481091. 39624840 10.3389/fphar.2024.1481091 PMC11608977

[34] Hu Z, Chen D, Yan P, Zheng F, Zhu H, Yuan Z, et al. Puerarin suppresses macrophage M1 polarization to alleviate renal inflammatory injury through antagonizing TLR4/MyD88-mediated NF-κB p65 and JNK/FoxO1 activation. Phytomedicine. 2024;132:155813. 10.1016/j.phymed.2024.155813. 38905846 10.1016/j.phymed.2024.155813

[35] Chambers JW, LoGrasso PV. Mitochondrial c-Jun N-terminal kinase (JNK) signaling initiates physiological changes resulting in amplification of reactive oxygen species generation. J Biol Chem. 2011;286(18):16052–62. 10.1074/jbc.M111.223602. 21454558 10.1074/jbc.M111.223602 PMC3091214

[36] Smith MJ, Fowler M, Naftalin RJ, Siow RCM. UVA irradiation increases ferrous iron release from human skin fibroblast and endothelial cell ferritin: consequences for cell senescence and aging. Free Radic Biol Med. 2020;155:49–57. 10.1016/j.freeradbiomed.2020.04.024. 32387586 10.1016/j.freeradbiomed.2020.04.024

[37] Du T, Song Y, Ray A, Chauhan D, Anderson KC. Proteomic analysis identifies mechanism(s) of overcoming bortezomib resistance via targeting ubiquitin receptor Rpn13. Leukemia. 2021;35(2):550–61. 10.1038/s41375-020-0865-2. 32424294 10.1038/s41375-020-0865-2 PMC7988682

[38] Zhang X, Yuan Z, Wu J, He Y, Lu G, Zhang D, et al. An orally-administered nanotherapeutics with carbon monoxide supplying for inflammatory bowel disease therapy by scavenging oxidative stress and restoring gut immune homeostasis. ACS Nano. 2023;17(21):21116–33. 10.1021/acsnano.3c04819. 37843108 10.1021/acsnano.3c04819

[39] Wang XD, Zhang JN, Gan YH, Zhou YH. Current understanding of pathogenesis and treatment of TMJ osteoarthritis. J Dent Res. 2015;94(5):666–73. 10.1177/0022034515574770. 25744069 10.1177/0022034515574770

[40] Zhao W, Zhang H, Liu R, Cui R. Advances in Immunomodulatory mechanisms of mesenchymal stem cells-Derived exosome on immune cells in Scar formation. Int J Nanomed. 2023;18:3643–62. 10.2147/IJN.S412717. 10.2147/IJN.S412717 PMC10327916 37427367

[41] Johnson J, Law SQK, Shojaee M, Hall AS, Bhuiyan S, Lim MBL, et al. First-in-human clinical trial of allogeneic, platelet-derived extracellular vesicles as a potential therapeutic for delayed wound healing. J Extracell Vesicles. 2023;12(7):e12332. 10.1002/jev2.12332. 37353884 10.1002/jev2.12332 PMC10290200

[42] Mondal J, Pillarisetti S, Junnuthula V, Saha M, Hwang SR, Park I-K, et al. Hybrid exosomes, exosome-like nanovesicles and engineered exosomes for therapeutic applications. J Control Release. 2023;353:1127–49. 10.1016/j.jconrel.2022.12.027. 36528193 10.1016/j.jconrel.2022.12.027

[43] Peng Y, Yang Z, Li J, Liu S. Research progress on nanotechnology of traditional Chinese medicine to enhance the therapeutic effect of osteoarthritis. Drug Deliv Transl Res. 2024;14(6):1517–34. 10.1007/s13346-024-01517-w. 38225521 10.1007/s13346-024-01517-w

[44] Guo J, Su K, Wang L, Feng B, You X, Deng M, et al. Poly(p-coumaric acid) nanoparticles alleviate temporomandibular joint osteoarthritis by inhibiting chondrocyte ferroptosis. Bioact Mater. 2024;40:212–26. 10.1016/j.bioactmat.2024.06.007. 38973989 10.1016/j.bioactmat.2024.06.007 PMC11224931

[45] Win S, Than TA, Fernandez-Checa JC, Kaplowitz N. JNK interaction with Sab mediates ER stress induced inhibition of mitochondrial respiration and cell death. Cell Death Dis. 2014;5(1):e989. 10.1038/cddis.2013.522. 24407242 10.1038/cddis.2013.522 PMC4040675

[46] O’Brien K, Tailor P, Leonard C, DiFrancesco LM, Hart DA, Matyas JR. Enumeration and localization of mesenchymal progenitor cells and macrophages in synovium from normal individuals and patients with pre-osteoarthritis or clinically diagnosed osteoarthritis. Int J Mol Sci. 2017. 10.3390/ijms18040774. 28379175 10.3390/ijms18040774 PMC5412358

[47] Fahy N, de Vries-van ML, Melle J, Lehmann W, Wei N, Grotenhuis E, Farrell PM, van der Kraan JM, Murphy YM, Bastiaansen-Jenniskens GJVM, van Osch. Human Osteoarthritic synovium impacts chondrogenic differentiation of mesenchymal stem cells via macrophage polarisation state. Osteoarthritis Cartilage. 2014;22(8):1167–75. 10.1016/j.joca.2014.05.021. 24911520 10.1016/j.joca.2014.05.021

[48] Jung D, Kim N-E, Kim S, Bae J-H, Jung I-Y, Doh K-W, et al. Plant-derived nanovesicles and therapeutic application. Pharmacol Ther. 2025;269:108832. 10.1016/j.pharmthera.2025.108832. 40023319 10.1016/j.pharmthera.2025.108832

[49] Yang S, Lu S, Ren L, Bian S, Zhao D, Liu M, et al. Ginseng-derived nanoparticles induce skin cell proliferation and promote wound healing. J Ginseng Res. 2023;47(1):133–43. 10.1016/j.jgr.2022.07.005. 36644388 10.1016/j.jgr.2022.07.005 PMC9834025

[50] Zhan W, Deng M, Huang X, Xie D, Gao X, Chen J, et al. *Pueraria lobata*-derived exosome-like nanovesicles alleviate osteoporosis by enhacning autophagy. J Control Release. 2023;364:644–53. 10.1016/j.jconrel.2023.11.020. 37967723 10.1016/j.jconrel.2023.11.020

[51] Wang X, Liu Y, Dong X, Duan T, Wang C, Wang L. peu-MIR2916-p3-enriched garlic exosomes ameliorate murine colitis by reshaping gut microbiota, especially by boosting the anti-colitic Bacteroides thetaiotaomicron. Pharmacol Res. 2024;200:107071. 10.1016/j.phrs.2024.107071. 38218354 10.1016/j.phrs.2024.107071

[52] Lu Y, Xu J, Tang R, Zeng P, Li Z, You J, et al. Edible *Pueraria lobata*-derived exosome-like nanovesicles ameliorate dextran sulfate sodium-induced colitis associated lung inflammation through modulating macrophage polarization. Biomed Pharmacother. 2024;170:116098. 10.1016/j.biopha.2023.116098. 38154276 10.1016/j.biopha.2023.116098

