# Supplementary material for: CAT/SOD-Enriched Achyranthes bidentata nanovesicles mitigate TMJOA via ROS scavenging and JNK/FOXO1 pathway Inhibition
Source: J Nanobiotechnology. 2025 Dec 23;23:782. doi: 10.1186/s12951-025-03834-9 (PMC12723957; doi:10.1186/s12951-025-03834-9)
Supplement: Supplementary file 1 — Supplementary Material 1 [file 12951_2025_3834_MOESM1_ESM.docx]

**Supplementary Figures**


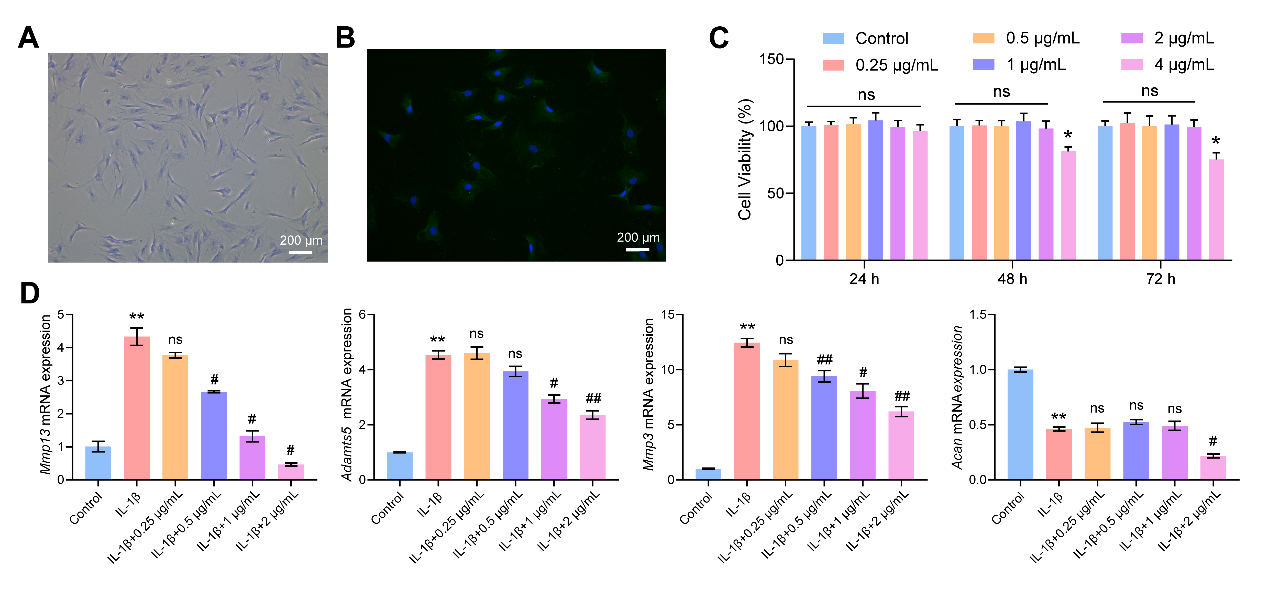


**Figure S1. Chondrocyte characterization and ABNV concentration optimization. (A)** Toluidine blue staining of primary condylar chondrocytes. **(B)** COL2A1 immunofluorescence confirming chondrocyte phenotype. **(C)** Viability of chondrocytes treated with ABNVs (0.25–4 μg/mL) for 24–72 h (CCK-8 assay). **(D)** qRT-PCR analysis of ECM-related genes (*Mmp13*, *Adamts5*, *Acan*) in IL-1β-stimulated chondrocytes treated with ABNVs (0.25–2 μg/mL). Data analyzed by one-way ANOVA (n=3); ^**^p<0.01 vs. Control; ^#^p<0.05, ^##^p<0.01 vs. IL-1β group; "ns" = not significant.


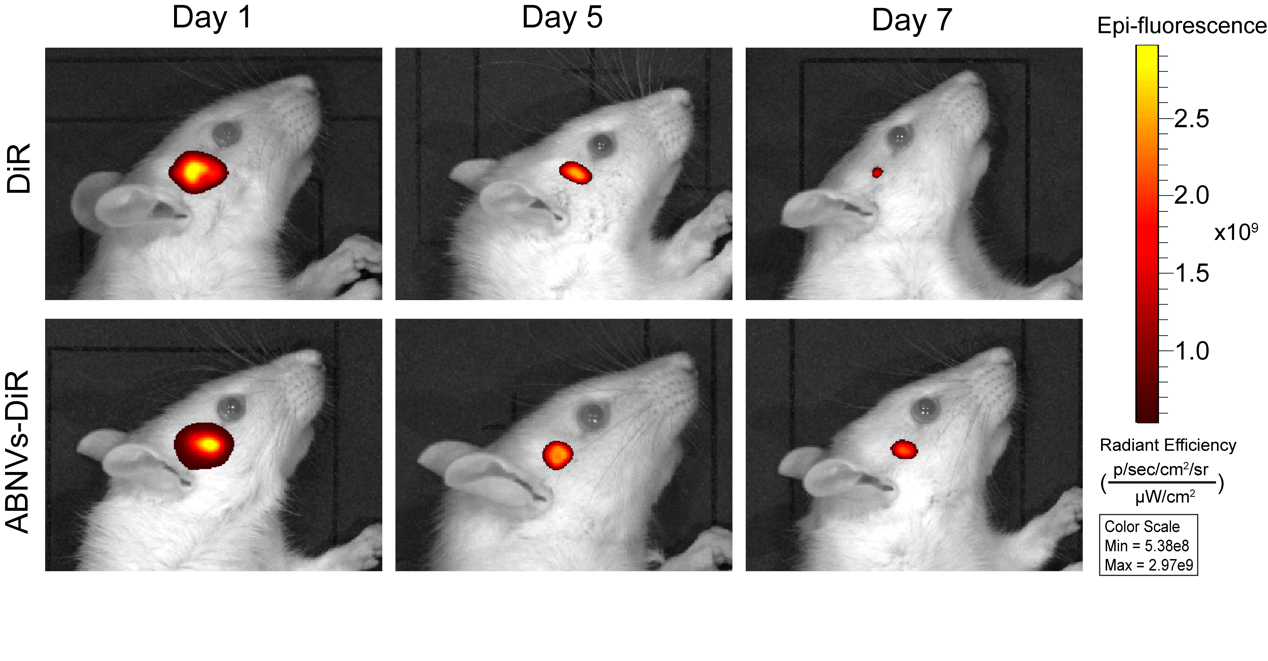


**Figure S2. IVIS images of the biodistribution of DiR and ABNVs-DiR.**


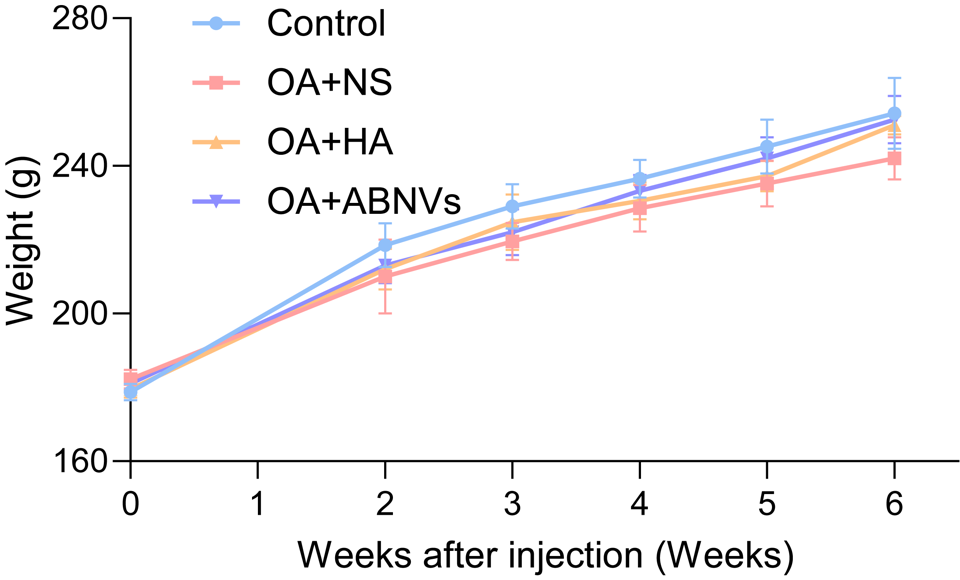


**Figure S3.** Changes in body weight of SD rats at different treatment stages (n=6).


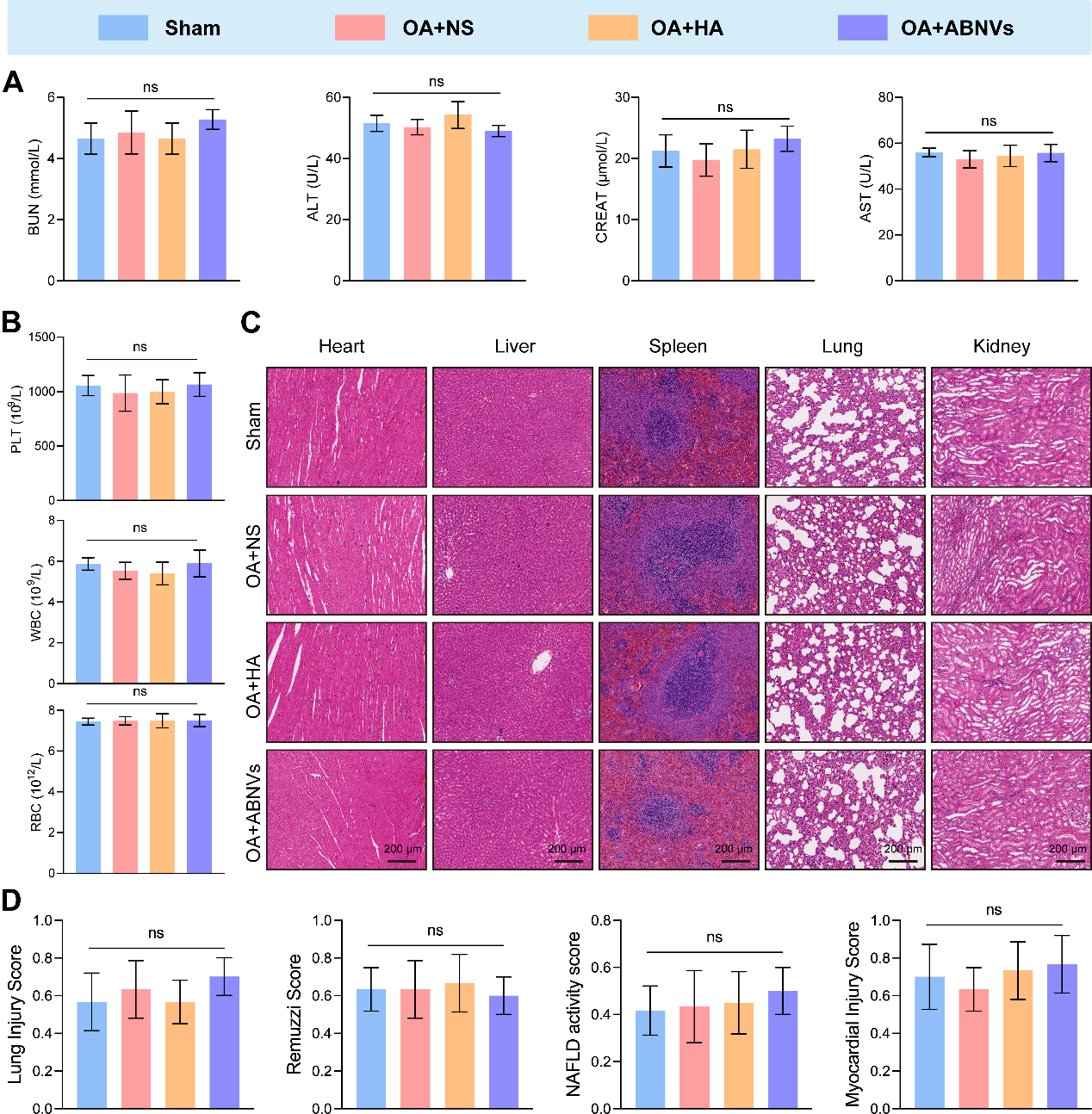


**Figure S4. Biosafety Analysis of Intra-articular Injection of ABNVs into Rats. (A)** Serum biochemistry, including BUN, CREAT, ALT, and AST. **(B)** Routine blood tests, including RBC, WBC, and PLT counts. **(C)** Histopathology and **(D)** histopathological grading of the major organs of rats following ABNVs treatment with HE staining; from left to right: heart, liver, spleen, lung, and kidney. Statistical analysis was conducted via one-way ANOVA (n = 3). Statistical significance is denoted as follows: ^*^*p* < 0.05 and ^**^*p* < 0.01 compared with the Sham group; ^#^*p* < 0.05 compared with the OA+NS group. "ns" indicates no significant difference.


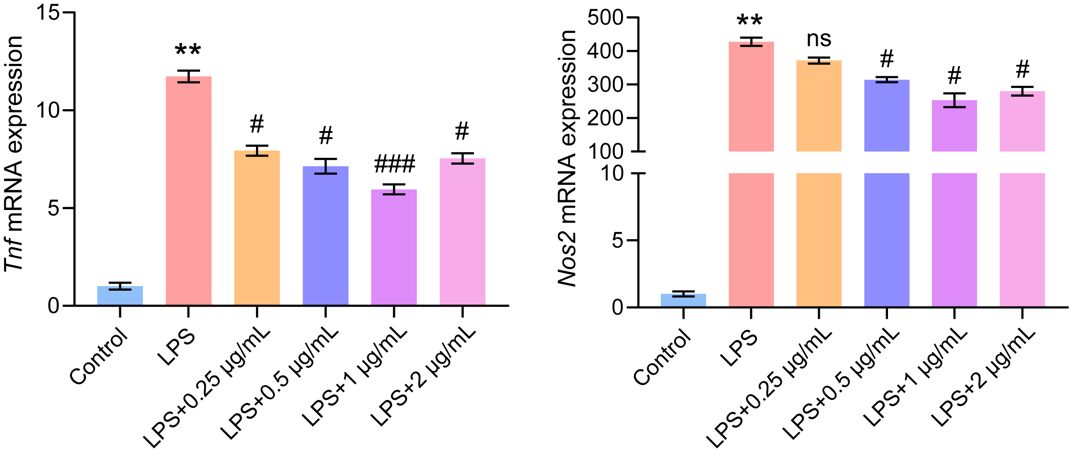


**Figure S5. Screening for suitable concentrations of ABNVs for in vitro experiments on macrophages.** (A) qRT-PCR analysis of different concentrations of ABNVs (0.25, 0.5, 1, or 2 μg/mL) on the alleviation of inflammatory indices after LPS stimulation of macrophages. Statistical analysis was conducted via one-way ANOVA (n=3). Statistical significance is denoted as follows: ^**^p < 0.01 compared with the Control group; ^#^p < 0.05 and ^###^p < 0.001 compared with the LPS group. "ns" indicates no significant difference.


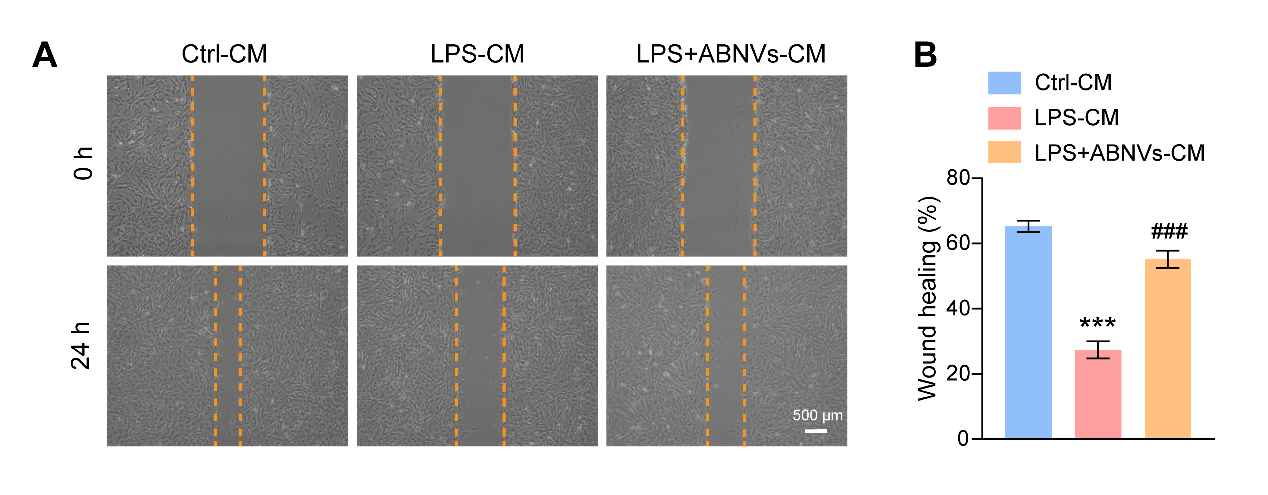


**Figure S6. ABNVs restore chondrocyte migration impaired by LPS-CM. (A)** Representative scratch assay images and **(B)** quantification showing migration of chondrocytes treated with Ctrl-CM, LPS-CM, or LPS+ABNVs-CM. Data analyzed by one-way ANOVA (n=3); ^***^p<0.001 vs. Ctrl-CM; ^###^p<0.001 vs. LPS-CM.


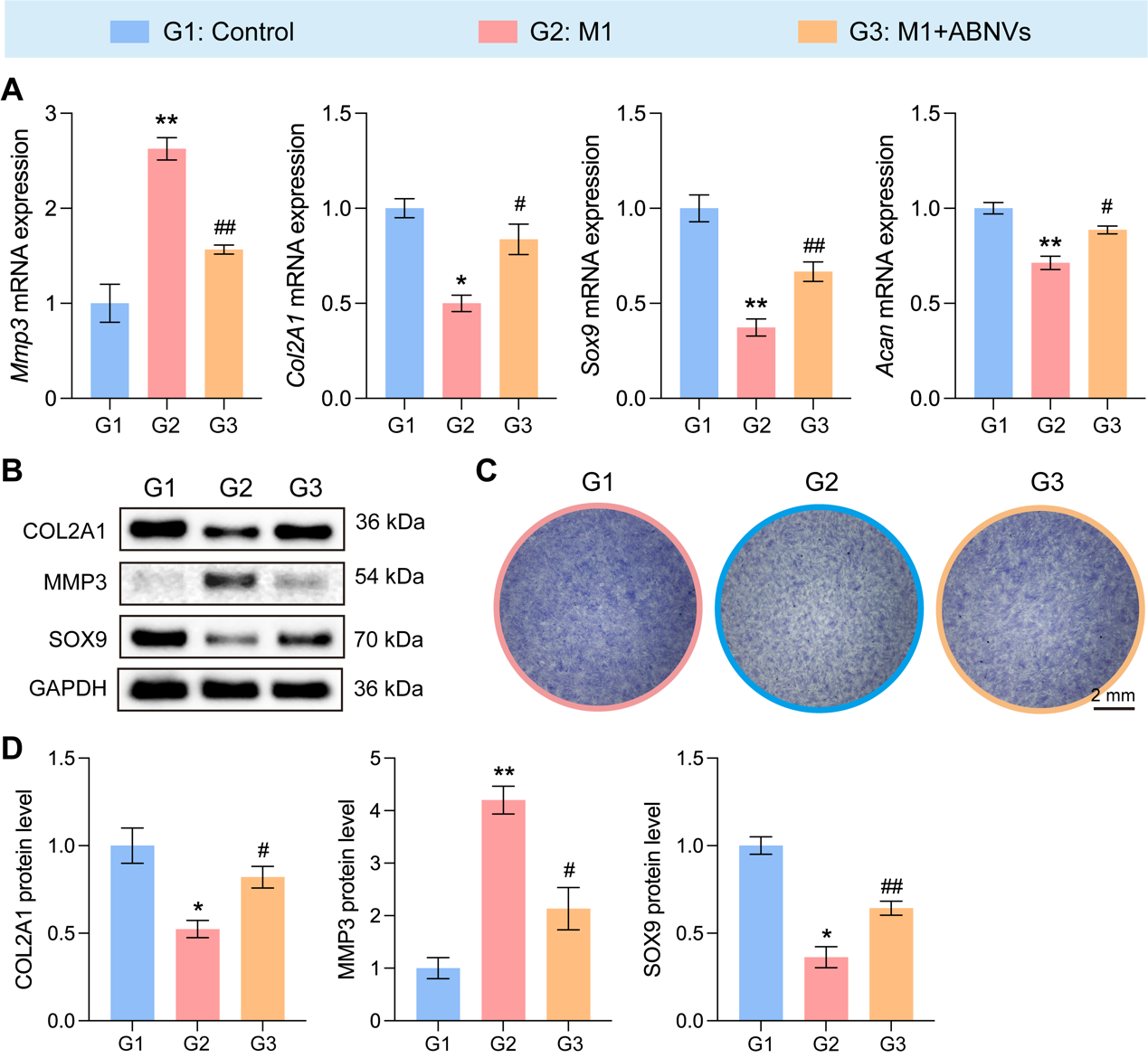


**Figure S7. ABNVs restore chondrocyte function by modulating macrophage responses.** **(A)** RT-qPCR of matrix genes (*Mmp3, Col2a1, Acan, Sox9*). **(B, D)** Western blot and quantification of SOX9, MMP3, and COL2A1. **(C)** Proteoglycan content (toluidine blue staining). Data analyzed by one-way ANOVA (n = 3); ^*^p < 0.05, ^**^p < 0.01 vs. Control; ^#^p < 0.05, ^##^p < 0.01 vs. M1 group.


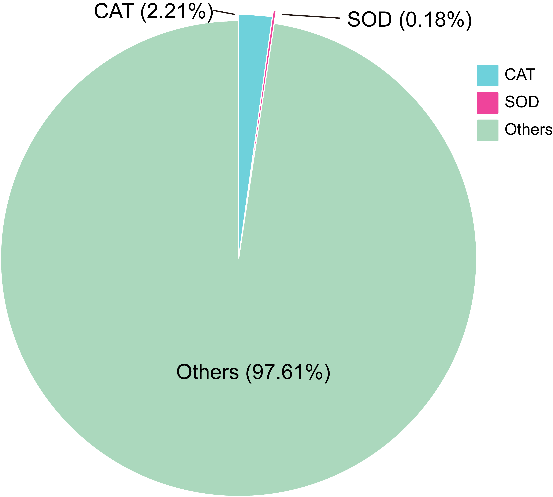


**Figure S8. Proteomics of Relative CAT and SOD Content in ABNVs.**


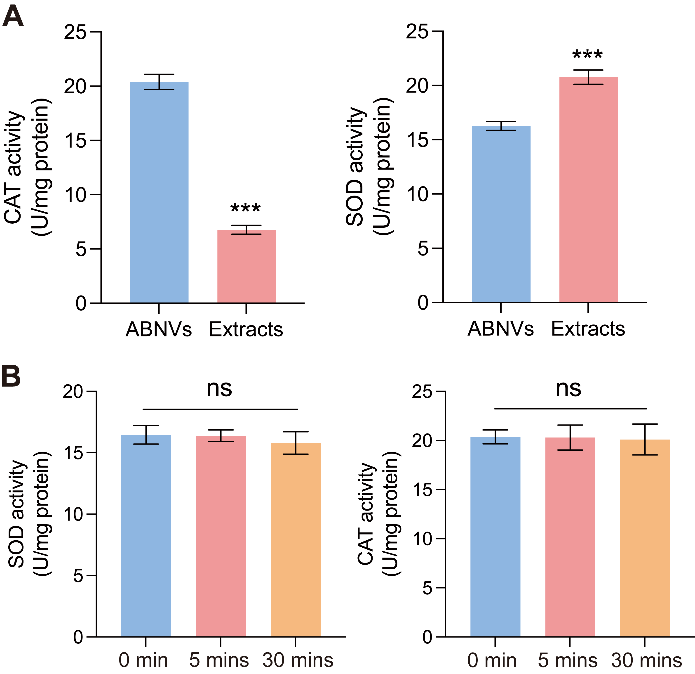


**Figure S9. Enzyme Activity Assays for CAT and SOD. (A)** Comparison of CAT and SOD Enzyme Activities Between ABNVs and Crude Extract. **(B)** Assay of CAT and SOD activity after trypsin digestion. Data analyzed by Student's t-test and one-way ANOVA (n=3); ^***^p<0.001 vs. ABNVs. "ns" indicates no significant difference.


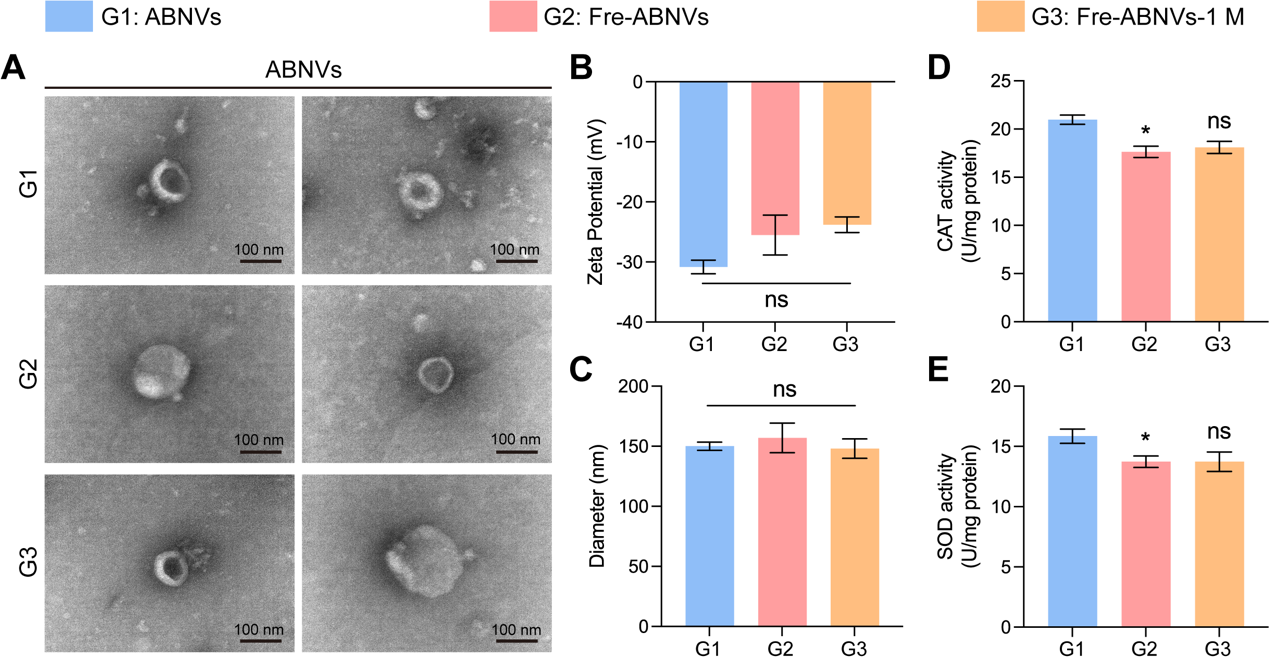


**Figure S10. Stability Testing of ABNVs After Freeze-Drying.** (A) TEM images of ABNVs. (B) The particle surface charge and size (C) of ABNVs were analyzed using NTA. (D, E) CAT and SOD enzymatic activity in ABNVs. Statistical analysis was conducted via one-way ANOVA (n = 3). Statistical significance compared with the ABNVs, ^*^p < 0.05. "ns" indicates no significant difference.


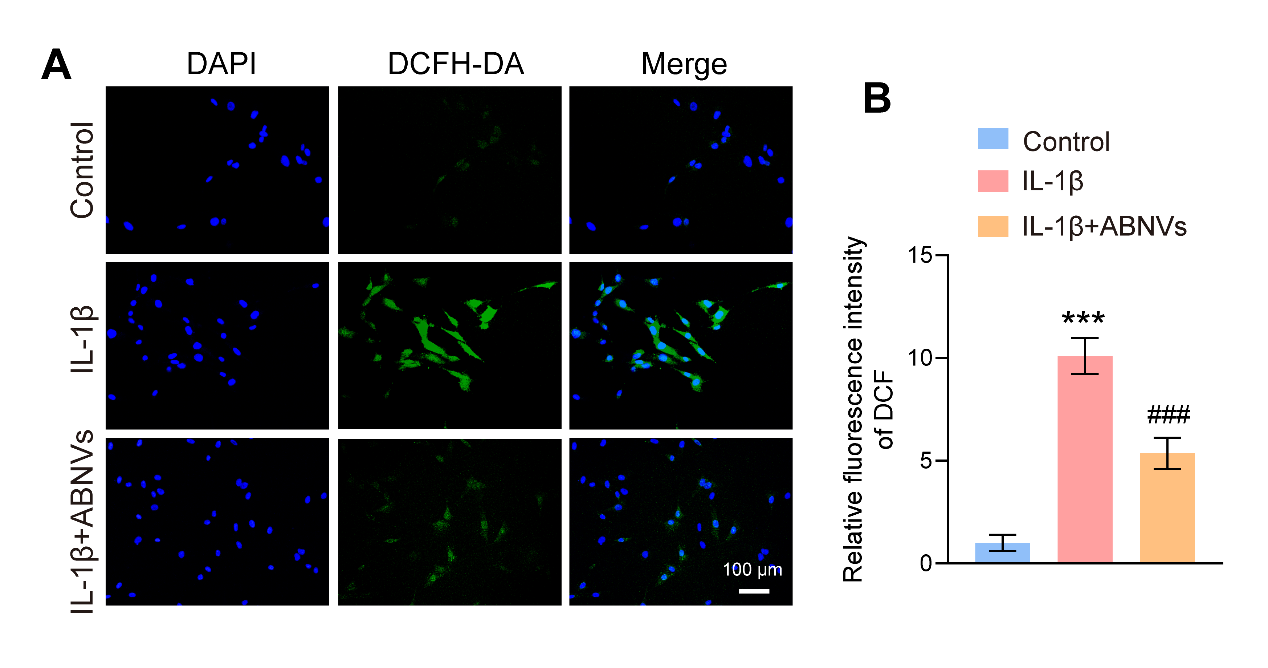


**Figure S11. ABNVs reduce ROS in chondrocytes. (A)** Fluorescence images and **(B)** quantification of intracellular ROS (DCFH-DA, green) in chondrocytes. Data analyzed by one-way ANOVA (n=3); ^***^p<0.001 vs. Control; ^###^p<0.001 vs. IL-1β group.


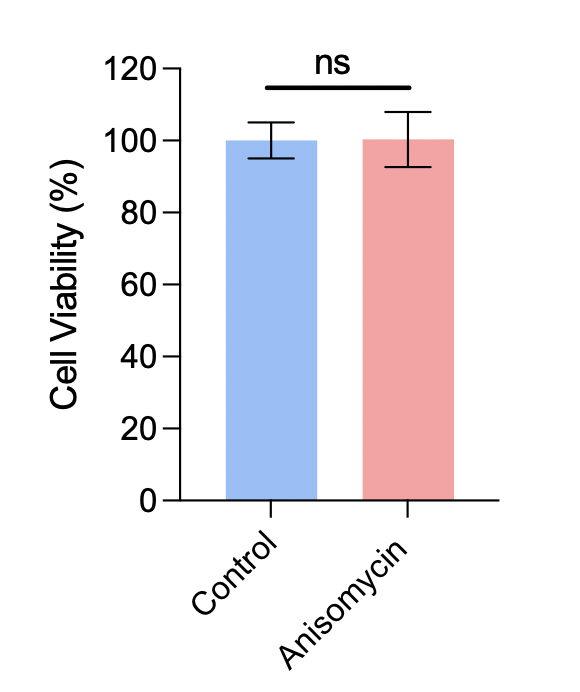


**Figure S12.** CCK-8 assay results after treating macrophages with anisomycin and replacing the culture medium for 24 hours. Data analyzed by Student's t-test (n=3); "ns" indicates no significant difference.


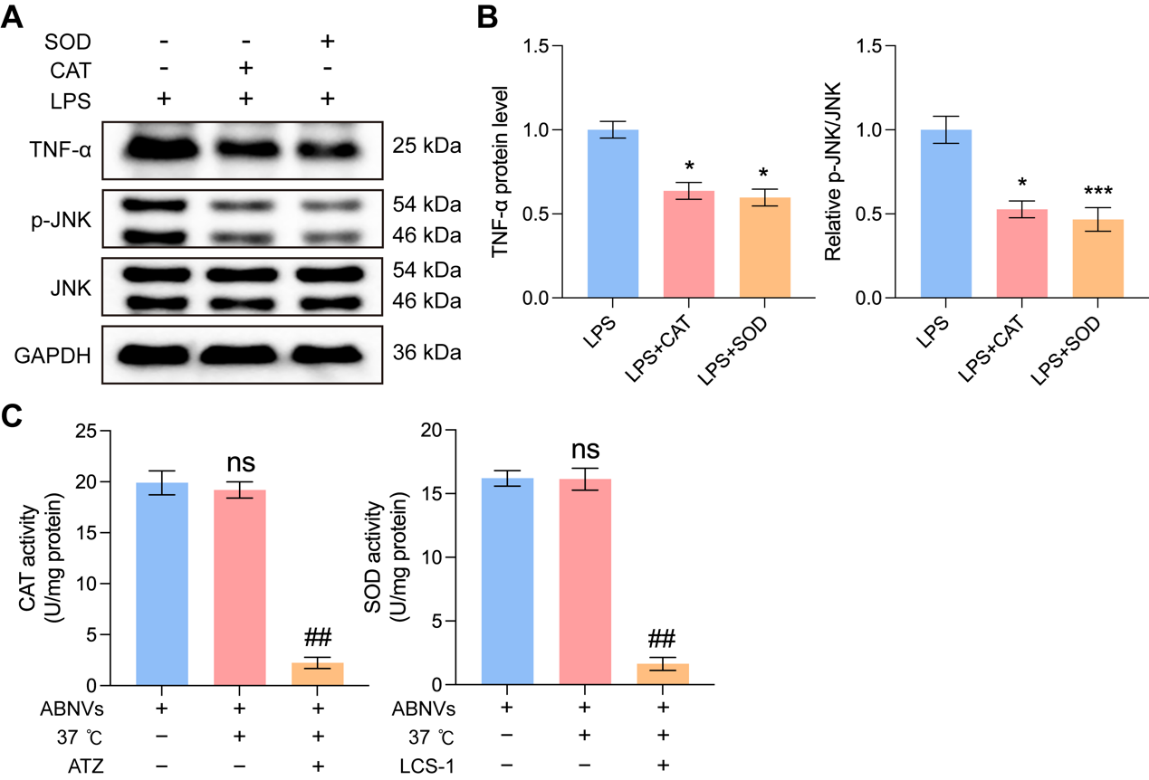


**Figure S13. (A)** Western blot results and **(B)** quantitative analysis of TNF-α and p-JNK/JNK in macrophages treated with exogenous SOD/CAT. **(C)** Enzyme Activity Assays for SOD and CAT. Data analyzed by one-way ANOVA (n = 3); ^*^p < 0.05, ^***^p < 0.001 vs. LPS; ^##^p < 0.01, vs. LPS-37℃ group. "ns" indicates no significant difference.


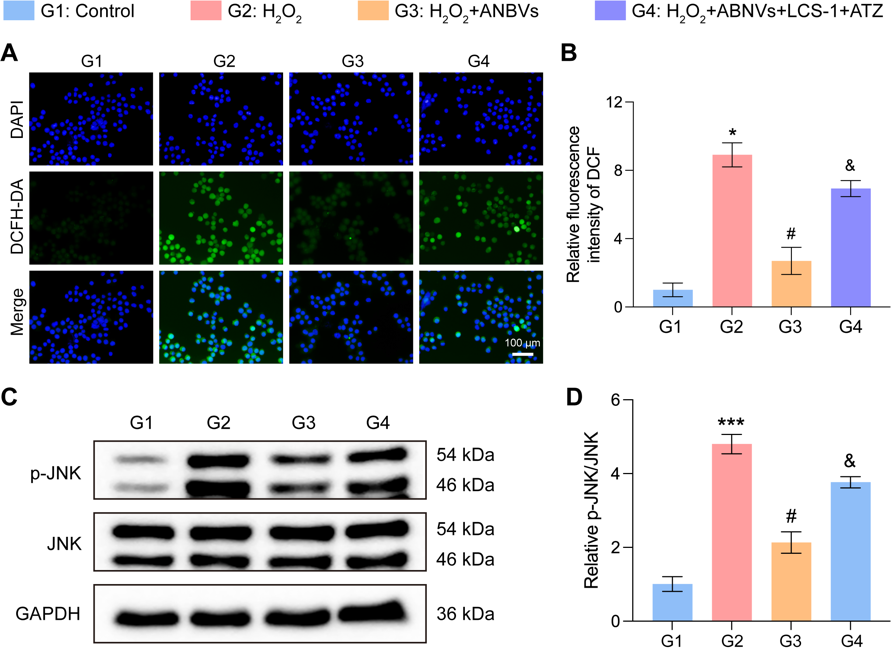


**Figure S14. ABNVs reduce hydrogen peroxide-induced ROS and p-JNK levels. (A, B)** Intracellular ROS levels (DCFH-DA staining, green) and quantification. **(C, D)** Western blot of p-JNK/JNK after LCS-1 (SOD inhibitor) and ATZ (CAT inhibitor) treatment. Data analyzed by one-way ANOVA (n=3); ^***^p<0.001 vs. Control; ^#^p<0.05 vs. H_2_O_2_; ^&^p<0.05, vs. H_2_O_2_+ABNVs.

**Table S1. Antibodies used for Western blot (WB), immunohistochemical (IHC), and immunofluorescence (IF).**

| **Antibodies** | **Manufacturer** | **Identifier** | **Application and Dilution** |
| --- | --- | --- | --- |
| rabbit anti-MMP3 | Abcam | ab52915 | 1:1000 for WB |
| rabbit anti-MMP-9 | Abcam | ab76003 | 1:1000 for WB |
| rabbit anti-Collagen II | Abcam | ab188570 | 1:1000 for WB |
| rabbit anti-SOX9 | CST | 82630T | 1:1000 for WB |
| rabbit anti-IL-6 | CST | 12912T | 1:1000 for WB |
| rabbit anti-TNF-α | Abcam | ab183218 | 1:1000 for WB |
| rabbit anti-FOXO1 | CST | 2880T | 1:1000 for WB |
| rabbit anti-phospho- FOXO1 | CST | 9461T | 1:1000 for WB |
| rabbit anti-SPAK/JNK | CST | 9252T | 1:1000 for WB |
| rabbit anti-phospho- SPAK/JNK | CST | 4668T | 1:1000 for WB |
| mouse anti-GAPDH | Proteintech | 60004-1-IG | 1:50000 for WB |
| rabbit anti-Collagen II | Servicebio | GB11021 | 1:100 for IHC |
| rabbit anti-IL-6 | Servicebio | GB11117 | 1:200 for IHC |
| rabbit anti-MMP13 | Servicebio | GB11247 | 1:200 for IHC |
| rabbit anti-Aggrecan | Servicebio | GB115687 | 1:500 for IHC |
| rabbit anti-Phospho-FOXO1 | Affinity | AF3417 | 1:100 for IHC |
| rabbit anti-Phospho-JNK | Affinity | AF3318 | 1:100 for IHC |
| rabbit anti-iNOS | Servicebio | GB11119 | 1:500 for IHC |
| rabbit anti-CD163 | Servicebio | GB113751 | 1:500 for IHC |
| rabbit anti-MMP3 | Servicebio | GB11131 | 1:400 for IF |
| rabbit anti-Collagen II | Servicebio | GB11021 | 1:300 for IF |
| rabbit anti-MMP9 | Servicebio | GB12132 | 1:500 for IF |

**Table S2. Primers used for RT-qPCR**

| **Gene** | **Acc.No.** | **Primer sequence (5’-3’)** | **Product length (bp)** |
| --- | --- | --- | --- |
| Mus GAPDH | NM_001411843.1 | F: GGCATCGAAGGTGGAAGAGT  R: AGGTGGTGAAGCAGGCATCT | 113 |
| Mus CD86 | NM_019388.3 | F: ACGTATTGGAAGGAGATTACAGCT  R: TCTGTCAGCGTTACTATCCCGC | 147 |
| Mus TNF-α | NM_013693.3 | F: CCTGTAGCCCACGTCGTAG  R: GGGAGTAGACAAGGTACAACCC | 148 |
| Mus IL-6 | NM_001314054.1 | F: CTGCAAGAGACTTCCATCCAG  R: AGTGGTATAGACAGGTCTGTTGG | 131 |
| Mus iNOS | NM 010927.4 | F: CAGAAGTGCAAAGTCTCAGACAT  R: GTCATCTTGTATTGTTGGGCT | 150 |
| Rat GAPDH | NM_017008.4 | F: CCATCAACGACCCCTTCATT  R: CACGACATACTCAGCACCAGC | 194 |
| Rat MMP3 | NM_133523.3 | F: TGAAGATGACAGGGAAGC  R: CTGGAGAATGTGAGTGGG | 235 |
| Rat MMP13 | NM_133530.1 | F: TGACCCAGCCCTATCCCT  R: ACCCTCCATAATGTCATACCC | 244 |
| Rat Sox9 | NM_080403.3 | F: AAGAGGCCACCGAACAGACT  R: GACCCTGAGATTGCCCGGAG | 132 |
| Rat Aggrecan | NM_022190.2 | F: GACCTGTGTGAGATCGACCA  R: GGTCGGGAAAGTGGCGATAA | 82 |
| Rat Adamts5 | NM_198761.2 | F: GGTCAGTGTTCTCGCTCTT  R: GTTAGGTGGGCAGGGTAT | 144 |
| Rat Mmp9 | NM_031055.2 | F: CTTGAAGTCTCAGAAGGTGGATC  R: CGCCAGAAGTATTTGTCATGG | 135 |
